# Click & sea: using bioorthogonal click chemistry to visualize seaweed cell walls

**DOI:** 10.1093/aob/mcaf103

**Published:** 2025-06-10

**Authors:** Wendy F Delpont, Godfrey Neutelings, Zoë A Popper

**Affiliations:** Botany and Plant Science, School of Natural Science, College of Science and Engineering, University of Galway, Galway H91 TK33, Ireland; Univ. Lille, CNRS, UMR 8576—UGSF—Unité de Glycobiologie Structurale et Fonctionnelle, 59000 Lille, France; Botany and Plant Science, School of Natural Science, College of Science and Engineering, University of Galway, Galway H91 TK33, Ireland

**Keywords:** Cell wall, seaweed, polysaccharide, bioorthogonal click chemistry, strain-promoted alkyne–azide cycloaddition

## Abstract

**Objectives:**

The study of seaweed cell walls, including their metabolism and composition, is crucial to monitor and understand their adaptation to climate change. Microscopy-based techniques that facilitate studies of seaweed cell walls *in situ*, including staining and immunolabelling, exist but have significant limitations, including that only a few monoclonal antibodies have been developed towards seaweed cell wall components. Furthermore, not all seaweed cell wall components have been described fully. This makes *in situ* studies focused on the metabolism of seaweed cell walls particularly challenging. Here, we present a method for labelling seaweed cell walls by incorporating chemical reporters *in muro*, followed by their association with fluorophores by click chemistry.

**Methods:**

Two different species of seaweed, representing different groups, i.e. the red seaweed *Phycodrys rubens* and the green seaweed *Ulva* spp., were selected for their abundance on the Irish coast and because they have relatively thin tissues, a feature likely to facilitate uptake and labelling using monosaccharide reporters. We selected three different activated sugars (fucose, galactose and glucose analogues) on the basis that they are major components of seaweed polysaccharides. Small sections of the seaweeds were incubated with the activated sugars, and their uptake and incorporation were visualized by attachment to the fluorescent probe AF488 and imaged using confocal microscopy.

**Results:**

After incubation with the activated sugars, the seaweed cell walls, i.e. the contours of the cells and, to a lesser extent, some cell organelles, fluoresced at 517 nm (emission wavelength of AF488), suggesting that the seaweed had incorporated the activated sugars. Fiji software was used to remove non-specific fluorescence (autofluorescence of the seaweed and non-specific binding of the fluorochrome), with the final images suggesting good and specific incorporation of the fluorochrome by the seaweeds. More interestingly, the fluorescence was associated primarily with the cell walls, implying that the activated sugars were incorporated predominantly into cell wall components (most probably either polysaccharides or glycoproteins).

**Conclusion:**

To date, bioorthogonal click chemistry has not been applied to seaweeds, but it represents a useful tool for phycologists to gain a better understanding of seaweed cell wall composition and dynamics, for example, throughout the seaweed life cycle or in the face of stresses (biotic or abiotic), including those resulting from climate change.

## INTRODUCTION

The ability to look inside living tissue, specifically inside cells, has always intrigued researchers and has long been a subject of study. However, it can be difficult to recognize and differentiate the cell components. Increasingly sophisticated methods have been developed over the years, enabling the identification of specific molecules, from histological tissue staining in the 1770s (Alturkistani *et al.*, 2016) to immunofluorescence targeting specific parts of polysaccharides and proteins (Coons *et al.*, 1941) to protein labelling via genetics (Chalfie *et al.*, 1994) and, more recently, the integration of fluorescent tags into the genome via genome editing (Schwinn *et al.*, 2018).

Seaweeds are predominantly marine photosynthetic organisms. They are multicellular organisms typically occurring in coastal locations and organized into rock-fixed thalli, although some, such as *Sargassum*, may be pelagic. Some seaweeds can be very small, measuring a few centimetres, whereas others can be ≤70 m long. However, despite similarities, including habitat, multicellularity and the ability to photosynthesize and produce sulphated polysaccharides, seaweeds are not a single group but include several lineages. Seaweeds are enormously diverse and possess many distinct features. However, they are frequently grouped according to their pigmentation into one of three taxa: green (Chlorophyta), red (Rhodophyta) and brown (Phaeophyceae).

In this manuscript, we will focus on their cell wall compositions, a feature that is sufficiently diverse that it has been used as an important character to define individual taxa (Stace, 1989). The green seaweed cell wall is mainly composed of cellulose microfibrils (made up of D-glucose residues), making up 15–30 % of the total dry weight of the seaweed (Wahlström *et al.*, 2020). Hemicellulose structures, which play a cohesive role in the wall, are also found. The main hemicelluloses present in green seaweed are xyloglucans, mannans and β-(1–3)-glucans. Other polysaccharides, called ulvans, are found only in the Chlorophyta. Ulvans are sulphated polysaccharides composed mainly of L-rhamnose and glucuronate chains, to which D-xylose, iduronic acid, D-mannose, D-galactose and fucose residues may be added. Ulvans are made up of a disaccharide repeat, whose composition can vary. However, there are two main combinations of repeats: type A and type B. Type A is called ulvanobiuronic acid 3-sulphate and is made up of rhamnose-3-sulphate and glucuronic acid or iduronic acid. Type B is called ulvanobiuronic acid 3-sulphate and is made up of rhamnose-3-sulphate and xylose (Lahaye and Robic, 2007). Carbohydrates are stored as starch in green seaweed, like in land plants. This starch is formed of two polymers, amylose and amylopectin, composed of D-anhydroglucopyranose (Prabhu *et al*., 2019).

Other molecules, such as glycoproteins, including arabinogalactan proteins and extensins, are also present in the cell walls of green seaweed. Arabinogalactan proteins and extensins also occur in green land plants (Clarke *et al.*, 1979); however, green seaweed arabinogalactan proteins and extensins have structural features that are seaweed specific, such as a unique sugar residue profile and the presence of sulphated sugars that are not present in most land plants (Estevez *et al.*, 2009; Liu *et al.*, 2016; Přerovská *et al.*, 2021).

In red seaweed cell walls, cellulose microfibrils constitute only 1–10 % of the total dry weight of the seaweed (Ross, 1953) and may be replaced by either glucomannans or xylans, depending on the species. Like ulvans in the Chlorophyta (green seaweed), red seaweed possesses specific polysaccharides, carrageenans and agars. Carrageenans are linear sulphated galactans composed of repeat units of α-D-galactopyranose and β-D-galactopyranose residues. There are 15 different types of carrageenans known, based on their degree of sulphation and the location of sulphate side-groups. Nevertheless, only three carrageenans are commonly found: the ι- (iota), κ- (kappa) and λ- (lambda) carrageenans (Lahaye, 2001; Stiger-Pouvreau *et al.*, 2016). Agar is composed of two different types of molecules called agarose and agaropectin. Both molecules are made up of disaccharide repeat units composed of-D-galactopyranose and anhydro-3,6-α-L-galactopyranose (Usov, 2011). Agarose is a linear polysaccharide, and agaropectin is a branched polysaccharide. Some sulphate groups may be attached to the agaropectin structure, which confers a degree of sulphation to the molecule (Lahaye and Rochas, 1991). Red seaweed also stores carbohydrates in the form of starch. However, this starch is different from the starch found in green seaweed and land plants and is called floridean starch. Although floridean starch is composed of a highly branched succession of α-(1→4)-linked glucose units (Ball *et al.*, 2011), it differs from the starch found in land plants by its composition and degree of branching.

Seaweed cell walls, like to those of land plants, play important roles in cell structure, cell growth and the maintenance of cell homeostasis. The cell wall is also a physical barrier involved in interacting with the environment and its various stresses and in cell–cell communication (Kloareg and Quatrano, 1988; Popper *et al.*, 2011). As a result, their cell wall compositions are diverse (Table 1), and many seaweeds are commercially important, predominantly because of their constituent polysaccharides that are largely used for their gelling and viscosifying properties in biomedical, pharmaceutical, cosmetic (Cardozo *et al.*, 2007; Pereira, 2018), food and feed, agricultural (Craigie, 2011) and textile (Lomartire *et al.*, 2022) industries, and for biofuels (Sharmila *et al.*, 2021) and bioremediation (Ibrahim, 2011; Makkar *et al.*, 2016; Ścieszka and Klewicka, 2019; Leandro *et al.*, 2020). In 2023, the market for seaweed products was valued at 5.3 billion USD, and forecasts predict an average annual growth of 6.4 % to reach a total market of 7.3 billion USD by 2028 (Marketsandmarkets, 2023). Although used in a wide range of fields and carrying considerable weight in the world markets, the components and dynamics of seaweed cell walls are not well studied *in situ*.

**Table 1.** Main polysaccharides present in cell walls of different seaweeds (adapted from Popper *et al.*, 2011).

Polysaccharides	Chlorophyta (green seaweed)	Rhodophyta (red seaweed)
Crystalline phase: fibre polysaccharides	Cellulose	Cellulose
Hemicellulose	Xyloglucan Fucan Mannan Glucuronan β-(1→3)-Glucan	Glucomannan Mannan β-(1→3)-Glucan β−(1→ 4)-Glucan
Amorphous polysaccharides	Ulvan	Agar Carrageenan Porphyran β−(1→3)-Galactan α−(1→3)-Glucan

Different techniques have been developed to study the cell walls and their components in seaweed. Specific histological stains, including Alcian Blue, Calcofluor White and Concanavalin A, can be used to observe and follow the metabolism of cell wall components. For example, Martone (2010) observed growth of the red seaweed *Calliarthron cheilosporioides* using Calcofluor White. Other techniques, such as immunolabelling and the development of specific monoclonal antibodies, for example, to different alginate structures, have allowed researchers to look at the development of zygotes in the brown seaweed *Fucus* (Torode *et al.*, 2016).

Despite the progress made in understanding seaweeds using these methods, there are many limitations, including that seaweed cell walls are highly diverse and that their components, even some of those that are amongst the most widely used, such as fucoidans, have not yet been fully characterized. A few monoclonal antibodies that were developed against land plant cell wall components can recognize features present in brown seaweeds (Raimundo *et al.*, 2016) and green seaweeds (Estevez *et al.*, 2009; Fernández *et al*., 2010). However, the development of highly specific monoclonal antibodies that label structures present in seaweed cell wall polysaccharides, giving insight into their location and metabolism, has been very limited. This is especially the case in comparison to the large array of different monoclonal antibodies available against plant cell wall components (Pattathil *et al.*, 2010; Duffieux *et al.*, 2020). Currently, only a few monoclonal antibodies are available that have been developed specifically against seaweed cell wall components. These include the range of monoclonal antibodies, commercially available from SeaProbes (https://www.sb-roscoff.fr/en/seaprobes), that recognize and bind to alginates and fucoidans found in brown seaweed (Torode *et al.*, 2015, 2016). These antibodies recognize alginates with different sulphation levels and that possess different ratios of glucuronic and mannuronic acids (Torode *et al.*, 2015, 2016) and have enabled improved knowledge of zygote development in brown seaweed (Torode *et al.*, 2015, 2016). Monoclonal antibodies have also been made to ulvans (Rydahl *et al.*, 2017). Many more seaweed cell wall polysaccharide-specific monoclonal antibodies have been developed, predominantly to brown seaweed polysaccharides (Vreeland, 1970; Jones *et al*., 1988; Vreeland and Laetsch, 1989) but also to carrageenans from red seaweeds (Vreeland *et al.*, 1992). Unfortunately, the majority of these antibodies are no longer commercially available to the scientific community (Raimundo *et al*., 2016), and information regarding the localization and dynamics of seaweed cell wall polysaccharides, although increasing in recent years (Estevez *et al.*, 2009; Fernández *et al*., 2010; Torode *et al.*, 2015, 2016; Raimundo *et al*., 2016), remains scarce (Raimundo *et al*., 2016), particularly for red seaweeds.

Red seaweed cell walls, despite the significant commercial importance of their polysaccharides, are comparatively less well characterized than green and brown seaweed cell walls, and there remains a distinct lack of available tools for studying their *in situ* dynamics, e.g. in response to climate change.

Huisgen (1961) developed the concept of 1,3-dipolar cycloadditions, which greatly inspired K. B. Sharpless, who first used the term ‘click chemistry’ (Kolb *et al.*, 2001). Researchers in his laboratory and that of Morten Meldal have developed copper-catalysed regioselective chemical associations of the Huisgen cycloaddition of azides and terminal alkynes to form 1,2,3-triazoles. They were awarded the Nobel Prize in Chemistry in 2022, along with Carolyn Bertozzi, who, in the late 1990s, described the possibility of carrying out such reactions in biological conditions inside cells. These reactions are called bioorthogonal, meaning that they occur rapidly in aqueous solutions, in the presence of biological molecules, at low concentrations, and are extremely selective, because they react with only a single functional group. The reactions resulting from these works are azide–alkyne cycloadditions depending [copper-catalysed azide–alkyne cycloaddition (CuAAC)] or not [strain-promoted alkyne–azide cycloaddition (SPAAC)] on copper. In the first case, the copper(I) ion lowers the activation energy, making the reaction highly efficient and selective, and in the second case, the strain in the cyclooctyne drives the reaction with the azide, bypassing the need for copper (Agard *et al*., 2004). The use of click chemistry approaches is generally straightforward from a technical point of view, with the only possible difficulties being the mobility of reporters across cell interfaces and verification of their compatibility with cellular metabolism. The real challenge, however, remains their synthesis when they are not commercially available, as is the case for lignin precursors (Morel *et al.*, 2022).

Although CuAAC and SPAAC have been applied successfully to study plant cell walls (Anderson *et al.*, 2012; Lion *et al.*, 2017; Simon *et al.*, 2018; Morel *et al.*, 2022), they appear not to have been applied to seaweed cell walls. In this article, we adapted and applied the SPAAC technique to seaweed to label their cell wall components. The seaweed species were chosen because of their availability on the Irish coast, meaning that the material used could be as fresh (and as metabolically active) as possible, and for the thinness of their tissues, which is typically associated with better uptake and integration of the reagents used in the click chemistry reactions. The green seaweed, *Ulva*, was also chosen because it has been sequenced (De Clerck *et al.*, 2018) and its cell wall components are relatively well characterized (Lahaye and Robic, 2007; Bothwell *et al.*, 2022). The red seaweed, *Chondrus crispus*, has also been sequenced (Collén *et al.*, 2013) and has commercial importance, in part derived from its high concentration of carrageenans. The gametophyte and sporophyte can also be distinguished, because the gametophyte generation exhibits autofluorescence (Fournet *et al.*, 1993).

To the best of our knowledge, click chemistry has not previously been applied to seaweed. We tested the integration of three modified sugar residues that commonly occur in cell wall components (fucose, galactose and glucose). We were then able to observe the cell components that had integrated the modified sugar residues by reacting them to attach a fluorescent dye and visualizing them using confocal microscopy.

## MATERIALS AND METHODS

### Collection of seaweed

Seaweeds were collected at low tide in May 2023 at An Spidéal, Co. Galway, Ireland (53°14′33″N, 9°18′23″W). This collection site is predominantly sandy, surrounded by sandstone rocks, in which there are intertidal pools at low tide.

**Fig. 1.**
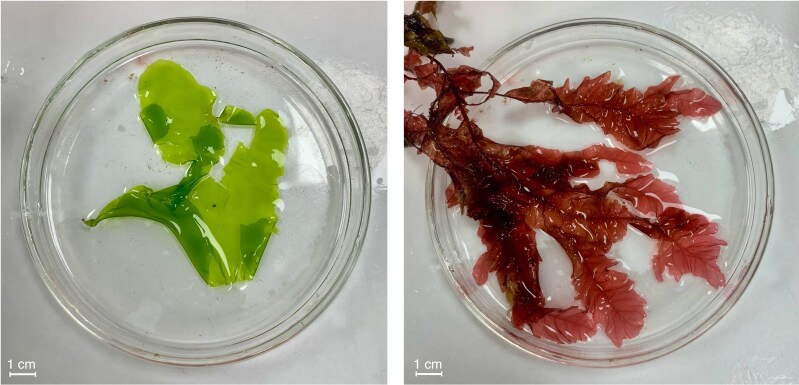
Photograph of *Ulva* spp. (left) and *Phycodrys rubens* (right), illustrating the two seaweeds studied. Photograph taken by W.F.D., Galway, Ireland, October 2023.

Two species of seaweed were collected: one red seaweed (*Rhodophyta*), *Phycodrys rubens*, and one green seaweed (*Chlorophyta*) *Ulva* spp. (Fig. 1). All the seaweeds collected were visually ungrazed or damaged, adult in appearance and clean, with no visible parasites.

After collection, the seaweed samples were transported to the laboratory in a sealed plastic bag containing seawater. In the laboratory, the samples were washed using clean seawater to remove any particles of sand and placed into 50 mL Falcon tubes containing clean seawater. The samples were stored at 10 °C for ∼20 h, followed by storage at 4 °C for 40 h. The seaweed samples were then cut into small square sections (∼20 mm × 20 mm) using dissecting scissors.

### Sugar reporters

**Fig. 2.**
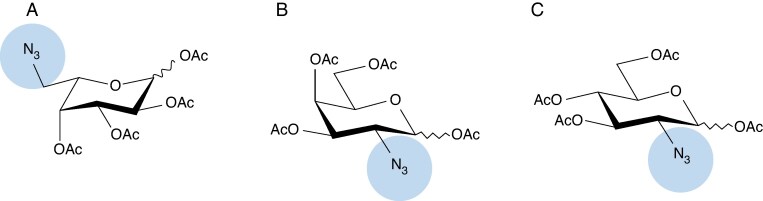
Structure of azido activated sugar residues used as reporters of sugar residues present in seaweed cell wall polysaccharides: (A) 1,2,3,4-tetra-*O*-acetyl-6-azido-L-fucopyranose; (B) 1,3,4,6-tetra-*O*-acetyl-2-azido-2-deoxy-D-galactopyranose; and (C) 1,3,4,6-tetra-*O*-acetyl-2-azido-2-deoxy-D-glucopyranose. The azide part, shown in the circle, can bind with an alkyl part (e.g. shown in Fig. 3) that can be used to visualize the sugars.

The ability of the seaweed sections to take up and incorporate three common monosaccharide reporters was tested. The monosaccharide reporters were 1,2,3,4-tetra-*O*-acetyl-6-azido-L-fucopyranose (TF608, Synthose, Concord, ON, Canada), 1,3,4,6-tetra-*O*-acetyl-2-azido-2-deoxy-D-galactopyranose (TL709, Synthose) and 1,3,4,6-tetra-*O*-acetyl-2-azido-2-deoxy-D-glucopyranose (TG713, Synthose) (Fig. 2). These three modified monosaccharides contain an azide moiety, which allows another molecule, previously linked by an alkyl moiety, to attach to them together and form a triazole. In addition, the peracetylated forms were chosen to allow transport inside the cells (Anderson and Wallace, 2012).

### Fluorochrome

**Fig. 3.**
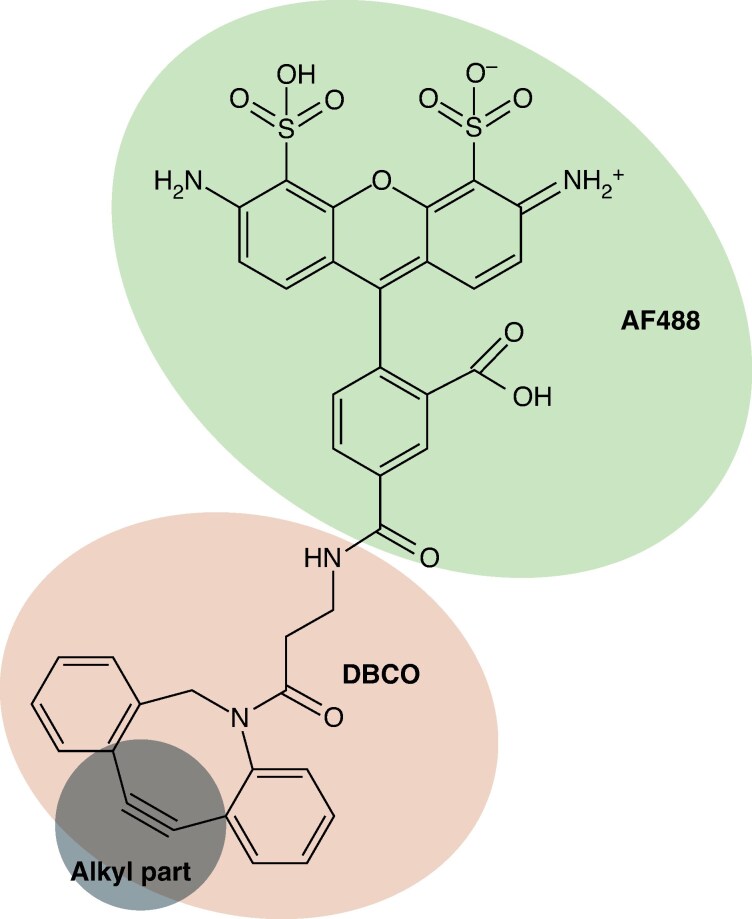
Structure of dibenzocyclooctyne-Alexa Fluor 488 (DBCO-AF488). The AF488 part is shown in the top circle and the DBCO part in the middle circle. The alkyl group, which can bind to the azide group on the modified sugar reporter, is shown in the lower circle.

For the modified sugars to bind to an azide group, a molecule containing an alkyl group must be present. To visualize the modified sugars, they must be linked to a probe that can be seen under the microscope. Fluorescent probes have typically been used for click chemistry (Mbua *et al.*, 2011; Subramanian *et al.*, 2014; Simon *et al.*, 2018). Dibenzocyclooctyne bound to the fluorescent probe AF488 (DBCO-AF488) was used in this study (Fig. 3). We chose to use AF488 to avoid interference from strong autofluorescence. Autofluorescence from seaweed would, most probably, be derived from chlorophyll *a*. Therefore, the selection of the fluorescent probe AF488 limits autofluorescence from chlorophyll *a*, because AF488 emits at a wavelength of 517 nm, i.e. in the green part of the spectrum, whereas chlorophyll *a* emits between 685 and 690 nm, in the red part of the spectrum.

### Reporter integration

**Fig. 4.**
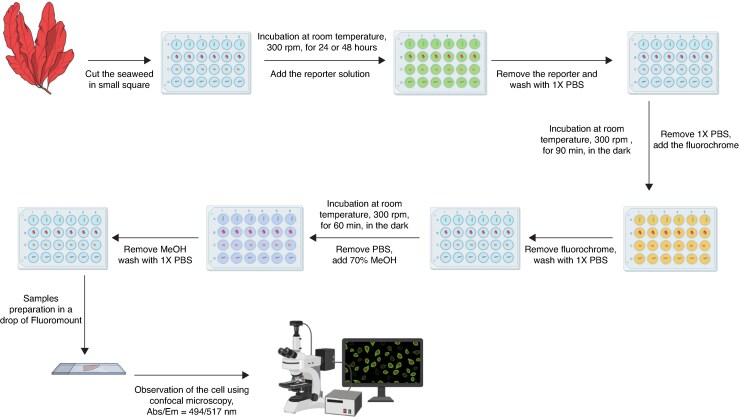
An illustrated summary of the bioorthogonal labelling strategy used in this work on seaweeds (created using BioRender.com within the agreement number SE27RFKPIS).

The experimental protocol is summarized in Fig. 4. Three fragments of each seaweed species were placed in the wells of 24-well microplates with 500 µL of 1× phosphate-buffered saline (PBS) solution. The seaweed sections were incubated in 300 µL of 10 mM of the reporter solution at room temperature, under agitation at 300 rpm for either 24 or 48 h. Two controls were carried out: (1) to determine autofluorescence of seaweed cell components using confocal microscopy [no reporter, no fluorochrome (R−, F−)]; and (2) non-specific binding of the fluorochrome [no reporter, plus fluorochrome (R−, F+)].

### Fluorochrome integration

After either 24 or 48 h, the reporter solution was removed, and the seaweed sections were washed three times using 500 µL of 1× PBS solution. The sections were then incubated at room temperature, in the dark, in 5 µm DCBO-AF488 (Jena Bioscience, Jena, Germany) solution for 90 min, with 300 rpm agitation (Thermo-Shaker, PHMP-4). After addition of the probe, all the following steps were carried out in the dark to limit the degradation of the probe.

### Tissue washing

Tissue washing was carried out by incubating the sections in methanol 70 % v/v for 1 h, in the dark, with 300 rpm agitation, in the dark. This step aimed to remove the fluorophores non-specifically bound to the cells and fix the cells to allow better observation. The samples were washed three times with 500 µL of 1× PBS solution.

### Confocal microscopy

Before observation, the samples were placed in a drop of Fluoromount-G^®^ (Thermo Fisher Scientific) between a slide and a coverslip and observed using a confocal microscope (Zeiss LSM 740). The excitation and emission wavelengths for Alexa Fluor^®^ 488-Azide were 494 and 517 nm, respectively. Microscope settings (gain, laser intensity and pinhole size) were kept constant throughout a given set of experiments to adjust all images in the same way and achieve comparable visualization of image fluorescence intensities. The images were captured, then analysed and processed using the Fiji software (Schindelin *et al.*, 2012). Each measurement was performed on three independent fragments.

### Supplementary analysis: validation of seaweed cell components

To gain a better understanding and provide additional analytical insights into the images obtained through click chemistry, particularly regarding the intracellular structure of seaweed, iodine and Toluidine Blue O (TBO) staining were performed after the observation of the click chemistry images. *Ulva* spp. and *Phycodrys rubens* were collected on 15 November 2024, at An Spidéal (Co. Galway, Ireland).

#### Iodine staining

Iodine staining (or Lugol test) was performed on plant cells to localize starch. However, cell wall polysaccharides, such as xyloglucans (Yuguchi *et al.*, 2005), carrageenans (which stain pale purple) and agars (Flint, 1990), can also be stained. No publications report the staining of ulvan by the Lugol test. However, xyloglucan is present in the cell walls of green seaweeds, which suggests that their cell walls might be stained by the Lugol test (Wahlström *et al.*, 2020).

Iodine staining is made possible by binding iodine and potassium iodide within sugar chains, specifically within the amylose helices of starch (Minick *et al.*, 1991). To carry out iodine staining, *Ulva* spp. and *Phycodrys rubens* sections were incubated with one drop of Lugol solution (Sigma Aldrich). After staining, the seaweed tissues were mounted on a microscope slide and covered with a coverslip prior to observation under a light microscope (Optika B-293 and camera Optika C-P6).

#### Toluidine Blue O staining

Toluidine Blue O (TBO) staining was performed to visualize the cell walls, chloroplasts and starch. The TBO staining method highlights acidic components in cells and tissues. TBO binds specifically to acidic groups, such as sulphates, carboxylates and phosphates, that are commonly found in polysaccharides, nucleic acids and glycoproteins (O’Brien *et al.*, 1964). To perform TBO staining, 0.05 % Toluidine Blue O powder was dissolved in 50 mm sodium acetate buffer (pH 5). A drop of the staining solution was applied to the seaweed tissue and incubated for 2 min at room temperature. The tissue was then rinsed with distilled water and mounted between a slide and coverslip for microscopic observation (Optika B-293 and camera Optika C-P6).

## RESULTS

### Effects of incubation time on labelling intensities

**Fig. 5.**
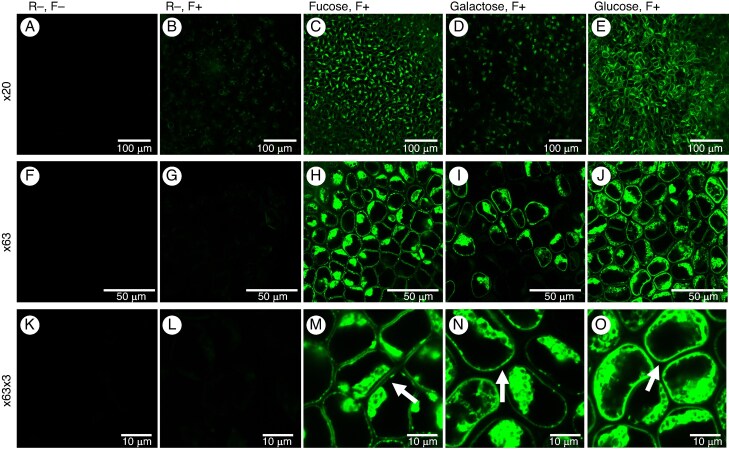
Labelling of green seaweed *Ulva* spp. tissues after 48 h incubation with various reporters. Reporters and fluorochrome: (A, F and K) no reporter (R−), no fluorochrome (F−) at ×20, ×63 and ×63 × 3 (×63 expanded three times), respectively; (B, G and L) (R−), with fluorochrome (F+) at ×20, ×63 and ×63 × 3 (×63 expanded three times), respectively; (C, H and M) fucose (R+) (F+) at ×20, ×63 and ×63 × 3, respectively; (D, I and N) galactose (R+) (F+) at ×20, ×63 and ×63 × 3, respectively; (E, J and O) glucose (R+) (F+) at ×20, ×63 and ×63 × 3, respectively. The arrows indicate the cell walls.

**Fig. 6.**
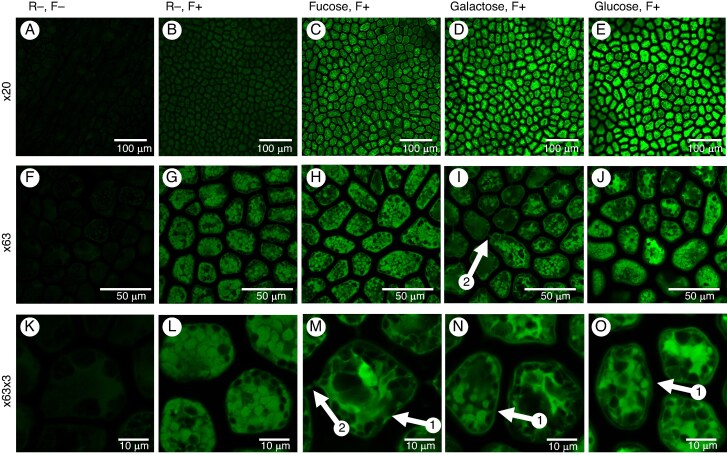
Labelling of tissues of the red seaweed *Phycodrys rubens* after 48 h incubation with various reporters. Reporters and fluorochrome: (A, F and K) no reporter (R−), no fluorochrome (F−) at ×20, ×63 and ×63 × 3 (×63 expanded three times), respectively; (B, G and L) (R−), with fluorochrome (F+) at ×20, ×63 and ×63 × 3 (×63 expanded three times), respectively; (C, H and M) fucose (R+) (F+) at ×20, ×63 and ×63 × 3, respectively; (D, I and N) galactose (R+) (F+) at ×20, ×63 and ×63 × 3, respectively; (E, J and O) glucose (R+) (F+) at ×20, ×63 and ×63 × 3, respectively. The arrows 1 indicate the cell walls; arrows 2 show the pit plugs.

No specific protocols have been established previously for chemical reporter detection in seaweed. We, therefore, incubated fragments of *Ulva* spp. and *Phycodrys rubens* in 10 µm N_3_-reporters (fucose, galactose and glucose) for 24 and 48 h without any permeabilizing agent to estimate the optimal time for incorporation. The overall results are summarized in Table 2. For *Ulva*, the 48 h incubation suggests that the longer the incubation, the greater the signal (Fig. 5). This is particularly correlated with more distinct labelling of the cell walls. In *Phycodrys*, the signal intensity also increased over time, but in the case of fucose, the signal intensity within the cell wall remained low at 24 and 48 h (Fig. 6; images of 24 h reporter incubation are available in Supplementary Data Figs S1 and S2).

**Table 2.** Comparison of the signal strength of AF488 for *Ulva* spp. and *Phycodrys rubens* incubated for either 24 or 48 h with the reporters for fucose (1,2,3,4-tetra-*O*-acetyl-6-azido-L-fucopyranose), galactose (1,3,4,6-tetra-*O*-acetyl-2-azido-2-deoxy-D-galactopyranose) or glucose (1,3,4,6-tetra-*O*-acetyl-2-azido-2-deoxy-D-glucopyranose) prior to visualization with DBCO-AF488 and imaging by confocal microscopy.

Conditions	Incubation time	*Ulva* spp.	*Phycodrys ruben*
R−/F+	24 h	Low signal	Moderate intracellular signal
	48 h	Low signal	Intracellular significant signal
Fucose/F+	24 h	Moderate signal, not diffused, labelled cell walls	Intense intracellular signal, slightly labelled cell wall
	48 h	High signal, diffused, labelled cell walls	Intense intracellular signal
Galactose/F+	24 h	Moderate signal, diffused, slightly labelled cell walls	Intense intracellular signal, low labelled cell walls
	48 h	High signal, not too diffused, labelled cell walls	Intense intracellular signal, slightly labelled cell walls
Glucose/F+	24 h	Moderate signal not diffused, labelled cell walls	Intense intracellular signal, low cell wall signal
	48 h	High signal, very diffused, intensely labelled cell walls	Intense intracellular signal, slightly labelled cell walls

### Incorporation of monosaccharide reporters in *Ulva* spp.

A first control lacking both reporters and probes (R−, F−) shows a total absence of signal in the thallus, indicating that there is no or undetectable autofluorescence of pigments or other cellular components at excitation/emission wavelengths of DCBO AF 488 (Fig. 5A, F, K). A second control incubated with only the fluorophore (R−, F+) exhibited a very weak fluorescent signal (Fig. 5B, G, L). This suggests that there is no strong non-specific binding of the probes.

The signal observed with the fucose reporter (fucose, F+) is intense (Fig. 5C, H, M) but does not appear to be uniform in all the cells (×20) compared with glucose labelling (Glucose, R+, F+), which appears to be more regular (Fig. 5E, J, O). The signal derived from the galactose reporter (Galactose, R+, F+) is visible in most of the seaweed cells and is more easily visualized at the higher magnifications, ×63 (Fig. 5I) and ×63 × 3 (Fig. 5N). Signals observed at high magnification reveal a strong signal for all the reporters at the level of the cell walls but also the inter-cell level.

It was possible to quantify and compare the signals obtained with the three reporters because, in each case, the same bioorthogonal click chemistry reaction occurred between an azide group on the reporter and DBCO anchored on the AF488 fluorophore. In addition, all the settings on the confocal microscope were conserved between each sample. Values were obtained by subtracting the background from the signal.

**Fig. 7.**
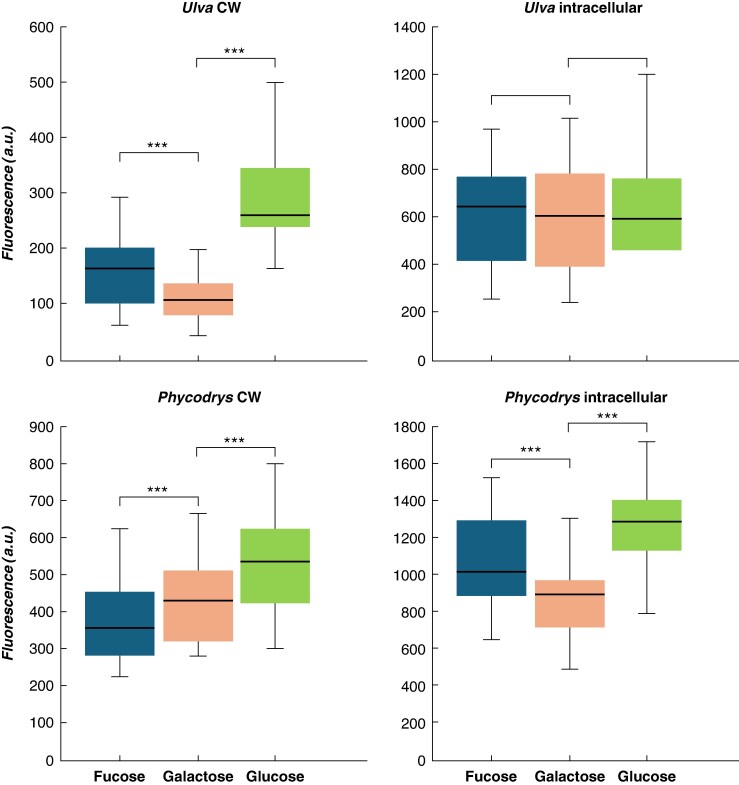
Incorporation of N_3_-labelled monosaccharides in red and green seaweed. Reporters were incorporated for 48 h and detected by the SPAAC reaction. Quantification was carried out by measuring fluorescence within the cell wall or inside the cell using FIJI, and a comparison was made between fucose, galactose and glucose surrogates. ***ANOVA (*P*-value <0.001).

Quantification was carried out on the cell walls and the internal space of the cells (Fig. 7). For *Ulva* spp., significant differences in incorporation levels were observed, with a higher rate for glucose, followed by fucose and, finally, galactose in the cell wall.

### Incorporation of monosaccharide reporters in *Phycodrys rubens*

Using the (R−, F−) control, it was possible to exclude the presence of autofluorescence in the *Phycodrys* samples (Fig. 6A, F, K). In contrast to the *Ulva* samples, however, we were able to observe the presence of non-specific fluorescence deriving from the presence of residual probes in the tissue, as suggested by the (R−, F+) control (Fig. 6B, G, L). In the (R+, F+) samples, the signal intensities were much higher (Fig. 6C–E, H–J, M–O). This suggests that the seaweed can specifically take up and incorporate the reporters. Each of the three different monosaccharides was located in the internal part of the cells, and, as for *Ulva* spp., it was interesting to observe the presence of clear labelling on the cell walls (Fig. 6M–O). In both compartments, the incorporation measured for glucose was the highest among the three reporters tested (Fig. 7). The comparison between galactose and fucose showed a higher signal for the first in the cell wall and the second inside the cells.

### Light microscopy of the intracellular structure of *Ulva* spp.

**Fig. 8.**
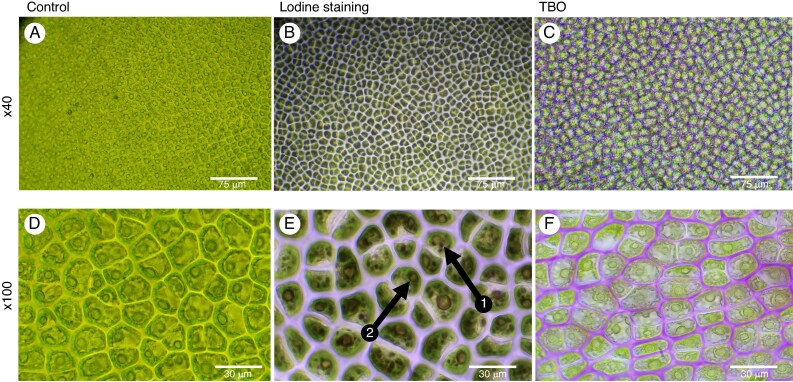
Tissues of the green seaweed *Ulva* spp. either unstained or stained with iodine or Toluidine Blue O. The tissues were unstained and observed at ×40 and ×100 (A and D, respectively) or stained with iodine (B and E) or TBO (C and F). Arrow 1 points to starch, and arrow 2 points to a nucleus.

Following the staining of *Ulva* spp. with Lugol’s solution, numerous black-staining spots ∼1–3 µm in diameter were observed (arrow 1; Fig. 8E). On average, around seven of these black-staining spots were present in each cell. Additionally, larger spots, ∼10–15 µm in diameter, surrounded by a brown-staining membrane, are visible (arrow 2; Fig. 8E). The cell walls are also visible, stained light brown (Fig. 8E).

TBO staining was also carried out on *Ulva*. The cell walls are stained pink/purple (Fig. 8F), indicating that they consist predominantly of polysaccharides. Additionally, structures with distinct contours can be observed inside the cells (Fig. 8F) and are likely to be cell organelles, including the vacuole, nucleus, mitochondria and peroxisome (Blomme *et al.*, 2021).

### Light microscopy of the intracellular structure of *Phycodrys rubens*

**Fig. 9.**
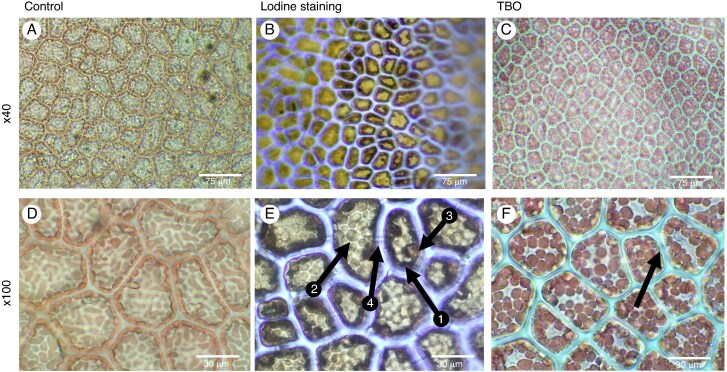
Tissues of the red seaweed *Phycodrys rubens* either unstained or stained with iodine or Toluidine Blue O. The tissues were unstained and observed at ×40 and ×100 (A and D, respectively) or stained with iodine (B and E) or TBO (C and F). Arrow 1 shows the chloroplast and other plastids, arrow 2 shows several chloroplasts, arrow 3 shows the nucleus, and arrow 4 shows the pit plugs.

Iodine staining (Lugol test) is known to stain floridean starch grains (Yu *et al.*, 2021). Iodine staining of *Phycodrys rubens* resulted in dark-staining spots ∼5–10 µm in diameter at the periphery of each cell in the stained tissue (arrow 1; Fig. 9E). There were approximately three brown-staining spots present in each cell. We also observed the presence of oval structures (5 µm long and 15 µm wide) that were lightly stained brown with Lugol’s stain (arrow 3; Fig. 9E). Therefore, there appear to be at least two distinct populations of cell organelles that stain with iodine in *Phycodrys*: (1) smaller, dark brown staining; and (2) larger organelles that are more lightly stained (arrow 3; Fig. 9E). Another structure is more clearly observed after iodine staining (arrow 4) and appears to be a connexion between two cells (Fig. 9F).

We also attempted to stain *Phycodrys* sections with Toluidine Blue O stain to visualize the cell walls, chloroplasts and starch. However, the red pigment, primarily phycobiliprotein, in *Phycodrys* masked any TBO staining; as a result, it was impossible to observe any distinct intracellular staining. In contrast, *Phycodrys* cell walls were stained blue–green and could be visualized clearly (Fig. 9F).

## DISCUSSION

### Using bioorthogonal click chemistry to monitor the production of cell wall components

We have explored the use of bioorthogonal click chemistry in two species of seaweeds (from the Chlorophyta and Rhodophyta phyla) by testing the metabolic incorporation of three different modified sugars, with the primary objective of incorporating them into cell wall polymers. Plant cell walls contain mainly polysaccharides and, when a secondary cell wall is present, also lignin. Each polymer is synthesized by enzymes located in different compartments, which can make the incorporation of chemical reporters more or less difficult. Lignin precursors, namely *p*-coumaryl alcohol, coniferyl alcohol and sinapyl alcohol, are synthesized in the cytoplasm and transported to the cell wall, where peroxidases and laccases oxidize them before they are incorporated into the growing polymer (Zhao *et al.*, 2013). To hijack these natural monomers, an exogenous supply of tagged surrogates on plant sections, meaning directly on the cell wall, is sufficient to induce the incorporation (Morel *et al.*, 2022). Concerning polysaccharides, metabolic labelling using sugar analogues compatible with click chemistry makes it necessary to set up conditions such that the reporters will enter the living cells through the cell wall and plasma membrane, then integrate the synthetic machinery in the Golgi apparatus for pectins and hemicelluloses or be activated in the cytoplasm for cellulose synthesis. This was first reported by the use of an alkynylated fucose analogue, which was incorporated successfully into the pectic rhamnogalacturonan-I within the cell walls of *Arabidopsis thaliana* roots (Anderson *et al.*, 2012). Rhamnogalacturonan-II labelling in this same organ but also in tobacco (*Nicotiana tabacum* L. cv Bright Yellow-2) cell suspension cultures was also demonstrated later on by using 8-azido-3-deoxy-D-manno-oct-2-ulosonic acid, an azido derivative of Kdo (Kdo-N3) (Dumont *et al.*, 2016). Kdo-N3 was also later incorporated into fast-growing, tip-polarized expanding pollen tubes (Ropitaux *et al.*, 2022).

Studies using click chemistry approaches have been carried out on microalgae in highly specific contexts. CuAAC click chemistry associated with liquid chromatography–mass spectrometry was used to detect polyether toxins (prymnesins) produced by *Prymnesium parvum*, which is associated with harmful seaweed blooms (Hems *et al.*, 2018). Other works have led to the production of microalgae-based microrobots functionalized by click chemistry with angiotensin-converting enzyme 2 (ACE2) receptor against the SARS-CoV-2 spike protein (Zhang *et al.*, 2021). But, to the best of our knowledge, the bioorthogonal click chemistry technique has never been reported in seaweed in the context of understanding biological mechanisms.

### Tracking analogues of monosaccharides in green and red seaweeds using a bioorthogonal click chemistry approach

*Ulva* spp., commonly known as sea lettuce, is an excellent model organism for studying green seaweed because of its simple and versatile life cycle and is a major component of marine food networks. It can form seaweed blooms (e.g. green tides) in response to nutrient pollution, providing a model to study eutrophication. It also exhibits high morphological plasticity, responding to environmental cues by altering its shape and structure (Wichard *et al.*, 2015). Galactose, fucose and glucose, tagged with azide groups, were detected after their association with a fluorescent probe by click chemistry. As described in the higher plant models, the peracetylated forms were able to cross the plasma membrane and were detected in different subcellular locations.

The strong labelling observed in the cell wall with the glucose reporter can be explained by the presence of (1) cellulose and (2) ulvans (structure detailed in the Introduction) within the cell wall of *Ulva*. Both these polymers are composed primarily of glucose residues, and their biosynthesis could incorporate the glucose reporter. In previous studies, fucose has been shown to represent the second-highest molar ratio in green seaweed monosaccharide composition, >20 %, in polysaccharides isolated from *Ulva linza* (formerly known as *Enteromorpha linza*) and *Ulva fasciata* (Zhang *et al.*, 2013; Barakat *et al.*, 2022). Ulvan polymers also contain low amounts of galactose, which was also shown to be present within *Ulva* cell wall glycoproteins (Estevez *et al.*, 2009).

The observation in *Ulva* of differences in labelling between the cells present in the same tissue (Fig. 5) could be explained by two reasons: (1) cells at different life stages, e.g. those that are entering senescence, would integrate the reporter less effectively; and/or (2) owing to the seaweed topology; although, superficially, the surface is relatively flat it, can be rugged in parts, and this would be likely to impact the uptake of the label.

The ultrastructure of *Ulva* has already been described. The seaweed is composed of two layers of cells, containing one chloroplast with multiple pyrenoids, a large vacuole and a thick cell wall (Messyasz *et al.*, 2013; Hu *et al.*, 2022). The results of the Lugol staining confirm the presence of starch inside the seaweed cells, as evidenced by the black-staining spots (Fig. 8E, arrow 1). In several cells, the nucleus is visualized as larger spots surrounded by a brown membrane (Fig. 8E, arrow 2). The cell walls are stained light brown owing to the presence of ulvan, a major polysaccharide in the cell walls of green seaweed, including *Ulva*.

These results correlate well with the observations made using click chemistry, where strong intracellular labelling was observed, particularly with the glucose reporter (Fig. 5E, J, O). This staining with iodine and labelling with the glucose reporter is likely to be associated with starch granules. Starch granules are composed of amylose and amylopectins, for which the major monosaccharide component is glucose (Fig. 5E, J, O;  Supplementary Data Fig. S1L) (Prabhu *et al*., 2019). However, it can be observed that glucose labelling in starch grains is not as bright as expected. This could be attributable to a stressful environment for the cells, affecting starch production (Zachleder and Brányiková, 2014).

For the fucose (Fig. 5C, H, M) and galactose (Fig. 5D, I, N) reporters, we hypothesize that the intracellular labelling observed represents cellular components involved in the synthesis of cell wall sugars, such as the endoplasmic reticulum and the Golgi apparatus. The peripheral localization of these components could also be explained by variations in osmotic pressure, which might influence their position within the cells. In *Ulva*, punctate fluorescence in the cell walls might be attributable to localized differences within the cell wall, resulting in the observed fluorescence pattern.

*Phycodrys rubens* is a perennial red seaweed belonging to the *Delesseriaceae* family, commonly found in the cold, temperate waters of the North Atlantic Ocean (Guiry and Guiry, 2024). It has a flattened, leaf-like thallus (body) that is typically reddish in colour. Its frond has a leafy appearance and is composed of a single flat layer of cells and can grow up to 3 cm wide. Analogues of galactose, fucose and glucose were incorporated into the cells, and the signals were detected inside the cells or in the cell wall. The labelling of cell walls by the galactose reporter is probably attributable to carrageenans and agars, which are polysaccharides specific to the cell walls of red seaweed. The galactose reporter might also label galactose-containing glycoproteins in the cell walls. Galactose was previously shown to be present in very high amounts (∼90 %) among the main neutral sugars identified in soluble dietary fibre from two red seaweeds, *Mastocarpus stellatus* and *Gigartina pistillata* (Gómez-Ordóñez *et al.*, 2014).

Although fucose is not a major monosaccharide component of red seaweed carbohydrates, several studies have reported its presence in these organisms. For instance, an antioxidant fucose-rich sulphated polysaccharide, additionally containing α-D-Man and β-D-Gal residues, was isolated from the red seaweed *Gloiopeltis tenax* (Lim and Ryu, 2009).

Iodine staining (Lugol reagent) effectively highlighted the presence of floridean starch in *Phycodrys* cells, as evidenced by the dark spots seen at the cell periphery (Fig. 9E, arrow 1), a feature previously described in the literature (Cole and Sheath, 1990). Similar structures are also visible in the control image (Fig. 9D). Arrow 2 shows the chloroplast inside the cytoplasm (Fig. 9E). The larger oval structures inside the cells (Fig. 9E, arrow 3), which were stained light brown, are likely to be nucleus, based on their staining properties (Meyer *et al.*, 2017). The structures observed between cells following staining with Lugol’s reagent and with TBO (Fig. 9E, arrow 4; Fig. 9F, arrow) appear to be the pit plugs, which are characteristic membranous structures specifically found in multicellular red seaweed. Pit plugs are also visible using fucose and galactose reporters in click chemistry (Fig. 6I, M and Supplementary Data Fig. S2E, arrow 2). Pit plugs facilitate cell–cell communication but are not considered homologous to plasmodesmata, which are found in land plants (Pueschel and Cole, 1982). However, despite their different origins, they perform similar roles.

We carried out TBO staining of *Phycodrys*. However, the presence of the red pigments, phycobilins and phycoerythrins, found associated with the chloroplast in many red seaweeds (French and Young, 1952; Gantt *et al.*, 2003), interfered with the visualization of the stain. The strong red coloration of the pigment made it difficult to distinguish intracellular features that might have stained with Toluidine Blue from the surrounding background. This limitation highlights the challenges when working with seaweed that contains strong pigments, such as phycoerythrin, which can mask other cellular details. However, blue–green staining of the cell wall with TBO could clearly be observed as a result of the binding of TBO to carrageenans that are highly sulphated (Gordon-Mills *et al.*, 1978). TBO stains *Ulva* cell walls a different colour because, despite the presence of sulphated groups in ulvans, they contain fewer sulphated groups than carrageenans.

There is a strong correlation between the intracellular components labelled by click chemistry, TBO and Lugol’s reagent. Click chemistry using activated fucose, glucose and galactose (Fig. 6M–O) highlights the presence of unlabelled structures localized at the periphery of the cells. These structures are also observed in the control using only the fluorochrome (Fig. 6L). Lugol staining allowed us to identify and confirm that these enigmatic structures are floridean granules.

Additionally, click chemistry enabled the labelling of intracellular structures in a non-specific manner, because these were visible both in the fluorochrome control (Fig. 6L) and with the activated sugars (Fig. 6M–O). These structures, similarly observed with Lugol and TBO staining, are likely to correspond to the seaweed phycobilisome complex, composed of the phycobiliprotein pigments involved in photosynthesis found in red seaweed (Gantt, 1980). Moreover, some of the intracellular structures were labelled by all of the activated sugar residues tested (Fig. 6M–O), suggesting that they could be either the location of carrageenans biosynthesis, i.e. within the Golgi apparatus, or carrageenans within transport vesicles (Vreeland *et al.*, 1992).

### A new experimental approach to characterize cell wall dynamics during seaweed growth

The possibility of incorporating monosaccharide-like chemical reporters into seaweed represents an opportunity to extend physiological and ecological studies of these aquatic species. Uniquely, this approach enables us to measure the potential of organisms to synthesize or modify their cell walls.

*Ulva* is highly adaptable to various environmental conditions, such as light and temperature fluctuations (Wu *et al.*, 2018). Interestingly, the morphotype of the thalli in *Ulva* can vary depending, in part, on the water salinity (Rybak, 2018). Indeed, tubular forms present in a very wide range of saline waters, whereas leaf-shaped thalli are found mainly in brackish waters. Given that organ phenotypes are dependent on cell shapes and organization within tissues, it is clear that approaches using chemical reporters associated with bioorthogonal chemistry will be highly relevant to the study of cell wall dynamics in these seaweeds.

One of the characteristics of Ulvaceae is their ability to produce large amounts of biomass over a short period of time (Rybak and Gąbka, 2018) in freshwater and even more markedly in seawater, where they are known to be the cause of green tides, for example, in the Yellow Sea off the coasts of Jiangsu and Shandong Province, China (Zhang *et al.*, 2019) or in the English Channel and Atlantic Sea off the coasts of Brittany (Louis *et al.*, 2023). Although this proliferation is largely attributable to an excess of nitrogenous nutrients, cell divisions require massive incorporation of sugars and, therefore, sustained production activities of cell wall materials that could be characterized by bioorthogonal click chemistry.

From a biotechnological point of view, the cell walls of seaweed could thus represent a target for better control of their proliferation. To achieve this, it is important to characterize the regulatory elements that enable their construction and then look for inhibitors. Several red seaweeds form blooms and cause premature mortality in marine animals as a result of toxin production (Young *et al.*, 2022). Here, too, knowledge of the wall-forming environment is a prerequisite for finding an effective solution. Additionally, red seaweeds are the primary source of agars and carrageenans, which are polysaccharides widely used as gelling, stabilizing and thickening agents in food, pharmaceuticals and cosmetics. Their production is greatly impacted by the photosynthetic activities of the organisms, but once the monosaccharides have been synthesized, the enzymatic machinery controlling their incorporation in the polymers remains the limiting factor. Bioorthogonal click chemistry can make it possible to select lines that perform better than others, in the same way as selection in conventional agriculture.

In summary, we were able to adapt the non-toxic SPAAC click chemistry technique on green and red seaweed. More specifically, we were able to label seaweed cell wall components reliably. Click chemistry is a technique with numerous advantages: it is relatively simple and inexpensive to perform. However, its interpretation is more complex than that of monoclonal antibodies, because the activated sugar can be integrated into various components of the seaweed. Although monoclonal antibodies recognize specific epitopes in polymers, their use is more limited. One advantage of click chemistry over antibody staining is its application in cases where polymers are not well defined (Popper, 2024).

This method opens many avenues of research, including the determination of changes in seaweed cell walls related to the life cycle and various stresses (grazing, pathogen infection, etc.) that could impact seaweed ecology and diversity, in addition to the quality of commercially grown seaweed and the products derived from them.

## Supplementary Material

mcaf103_Supplementary_Data

## SUPPLEMENTARY DATA

Figure S1: labelling of tissues of the green seaweed *Ulva* spp. after 24 h incubation with various reporters. Figure S2: labelling of tissues of the green seaweed *Phycodrys rubens* after 24 h incubation with various reporters.

## References

[mcaf103-B1] Agard N, Prescher J, Bertozzi C. 2004. A strain-promoted [3 + 2] azide–alkyne cycloaddition for covalent modification of biomolecules in living systems. Journal of American Chemical Society 126: 15046–15047. doi:10.1021/ja044996f 15547999

[mcaf103-B2] Alturkistani HA, Tashkandi FM, Mohammedsaleh ZM. 2016. Histological stains: a literature review and case study. Global Journal of Health Science 8: 72–79. doi: 10.5539/gjhs.v8n3p72 PMC4804027 26493433

[mcaf103-B3] Anderson CT, Wallace IS. 2012. Illuminating the wall: using click chemistry to image pectins in Arabidopsis cell walls. Plant Signaling & Behavior 7: 661–663. doi: 10.4161/psb.19939 22580708 PMC3442861

[mcaf103-B4] Anderson CT, Wallace IS, Somerville CR. 2012. Metabolic click-labeling with a fucose analog reveals pectin delivery, architecture, and dynamics in *Arabidopsis* cell walls. Proceedings of the National Academy of Sciences of the United States of America 109: 1329–1334. doi: 10.1073/pnas.1120429109 22232683 PMC3268317

[mcaf103-B5] Ball S, Colleoni C, Cenci U, Raj JN, Tirtiaux C. 2011. The evolution of glycogen and starch metabolism in eukaryotes gives molecular clues to understand the establishment of plastid endosymbiosis. Journal of Experimental Botany 62: 1775–1801. doi: 10.1093/jxb/erq411 21220783

[mcaf103-B6] Barakat KM, Ismail MM, Abou El Hassayeb HE, El Sersy NA, Elshobary ME. 2022. Chemical characterization and biological activities of ulvan extracted from *Ulva fasciata* (Chlorophyta). Rendiconti Lincei: Scienze Fisiche e Naturali 33: 829–841. doi: 10.1007/s12210-022-01103-7

[mcaf103-B7] Blomme J, Liu X, Jacobs TB, Clerck OD. 2021. A molecular toolkit for the green seaweed *Ulva mutabilis*. Plant Physiology 186: 1442–1454. doi: 10.1093/plphys/kiab185 33905515 PMC8260120

[mcaf103-B9] Bothwell JH, Goodridge AJ, Rapin M, et al **2022**. Cell walls are dynamically O-acetylated in the green seaweed, Ulva compressa. *bioRxiv* 493306. doi: 10.1101/2022.05.24.493306 [preprint: not peer reviewed].

[mcaf103-B10] Cardozo KHM, Guaratini T, Barros MP, et al 2007. Metabolites from algae with economical impact. Comparative Biochemistry and Physiology: Toxicology & Pharmacology: CBP 146: 60–78. doi: 10.1016/j.cbpc.2006.05.007 16901759

[mcaf103-B11] Chalfie M, Tu Y, Euskirchen G, Ward WW, Prasher DC. 1994. Green fluorescent protein as a marker for gene expression. Science 263: 802–805. doi:10.1126/science.8303295 8303295

[mcaf103-B12] Clarke A, Anderson R, Stone B. 1979. Form and function of arabinogalactans and arabinogalactan-proteins. Phytochemistry 18: 521–540. doi: 10.1016/S0031-9422(00)84255-7

[mcaf103-B13] Cole KM, Sheath RG. 1990. Biology of the red algae. Cambridge, UK: Cambridge University Press.

[mcaf103-B14] Collén J, Porcel B, Carré W, et al 2013. Genome structure and metabolic features in the red seaweed *Chondrus crispus* shed light on evolution of the Archaeplastida. Proceedings of the National Academy of Sciences of the United States of America 110: 5247–5252. doi: 10.1073/pnas.1221259110 23503846 PMC3612618

[mcaf103-B15] Coons A, Creech H, Jones R. 1941. Immunological properties of an antibody containing a fluorescent group. Experimental Biology and Medecine 47: 200–202. doi:10.3181/00379727-47-13084P

[mcaf103-B16] Craigie JS . 2011. Seaweed extract stimuli in plant science and agriculture. Journal of Applied Phycology 23: 371–393. doi: 10.1007/s10811-010-9560-4

[mcaf103-B17] De Clerck O, Kao S-M, Bogaert KA, et al 2018. Insights into the evolution of multicellularity from the sea lettuce genome. Current Biology: CB 28: 2921–2933. doi: 10.1016/j.cub.2018.08.015 30220504

[mcaf103-B18] Duffieux D, Marcus SE, Knox JP, Hervé C. 2020. Monoclonal antibodies, carbohydrate-binding modules, and detection of polysaccharides in cell walls from plants and marine algae. In: Popper ZA. ed. The plant cell wall: methods and protocols. New York, NY: Springer, 351–364. doi: 10.1007/978-1-0716-0621-6_20 32617945

[mcaf103-B19] Dumont M, Lehner A, Vauzeilles B, et al 2016. Plant cell wall imaging by metabolic click-mediated labelling of rhamnogalacturonan II using azido 3-deoxy-D-*manno*-oct-2-ulosonic acid. The Plant Journal: For Cell and Molecular Biology 85: 437–447. doi: 10.1111/tpj.13104 26676799

[mcaf103-B20] Estevez JM, Fernández PV, Kasulin L, Dupree P, Ciancia M. 2009. Chemical and in situ characterization of macromolecular components of the cell walls from the green seaweed *Codium fragile*. Glycobiology 19: 212–228. doi: 10.1093/glycob/cwn101 18832454

[mcaf103-B101] Fernández PV, Ciancia M, Miravalles AB, Estevez JM. 2010. Cell-wall polymer mapping in the coenocytic macroalga *Codium vermilara* (Bryopsidales, chlorophyta). Journal of Phycology 46: 456–465. doi: 10.1111/j.1529-8817.2010.00821.x 27020016

[mcaf103-B21] Flint OF . 1990. Micro-technique for the identification of food hydrocolloids. Analyst 115: 61–63. doi: 10.1039/AN9901500061

[mcaf103-B22] Fournet I, Deslandes E, Floc’h J-Y. 1993. Iridescence: a useful criterion to sort gametophytes from sporophytes in the red alga *Chondrus crispus*. Journal of Applied Phycology 5: 535–537. doi: 10.1007/BF02182512

[mcaf103-B23] French CS, Young VK. 1952. The fluorescence spectra of red algae and the transfer of energy from phycoerythrin to phycocyanin and chlorophyll. Journal of General Physiology 35: 873–890. doi: 10.1085/jgp.35.6.873 14938526 PMC2147322

[mcaf103-B24] Gantt E . 1980. Structure and function of phycobilisomes: light harvesting pigment complexes in red and blue-green algae. In: Bourne GH, Danielli JF, Jeon KW. eds. International review of cytology, Vol. 66. New York, NY: Academic Press, 45–80. https://www.sciencedirect.com/science/article/abs/pii/S0074769608619713

[mcaf103-B25] Gantt E, Grabowski B, Cunningham FX. 2003. Antenna systems of red algae: phycobilisomes with photosystem II and chlorophyll complexes with photosystem I. In: Green BR, Parson WW. eds. Light-harvesting antennas in photosynthesis. Dordrecht, Pays-Bas: Springer Netherlands, 307–322. doi: 10.1007/978-94-017-2087-8_10

[mcaf103-B26] Gómez-Ordóñez E, Jiménez-Escrig A, Rupérez P. 2014. Bioactivity of sulfated polysaccharides from the edible red seaweed *Mastocarpus stellatus*. Bioactive Carbohydrates and Dietary Fibre 3: 29–40. doi: 10.1016/j.bcdf.2014.01.002

[mcaf103-B27] Gordon-Mills EM, Tas J, McCandless EL. 1978. Carrageenans in the cell walls of *Chondrus crispus* stack. (rhodophyceae, gigartinales).: III. Metachromasia and the topooptical reaction. Phycologia 17: 95–104. doi: 10.2216/i0031-8884-17-1-95.1

[mcaf103-B28] Guiry MD, Guiry GM. 2024. Algaebase. World-Wide Electronic Publication, University of Galway. https://www.algaebase.org/ (21 June 2024, date last accessed).

[mcaf103-B29] Hems E, Wagstaff B, Saalbach G, Field R. 2018. CuAAC click chemistry for the enhanced detection of novel alkyne-based natural product toxins. Chemical Communications 54: 12234–12237. doi: 10.1039/C8CC05113E 30311608 PMC6243676

[mcaf103-B30] Hu M, Zhao S, Liu J, et al 2022. The morphology, genetic diversity, and distribution of *Ulva meridionalis* (Ulvaceae, Chlorophyta) in Chinese seas. Journal of Marine Science and Engineering 10: 1873. doi: 10.3390/jmse10121873

[mcaf103-B31] Huisgen R . 1961. 1,3-Dipolar cycloadditions. Proceedings of the Chemical Society 0: 357–396. doi: 10.1039/PS9610000357

[mcaf103-B32] Ibrahim WM . 2011. Biosorption of heavy metal ions from aqueous solution by red macroalgae. Journal of Hazardous Materials 192: 1827–1835. doi: 10.1016/j.jhazmat.2011.07.019 21798665

[mcaf103-B33] Jones JL, Callow JA, Green JR. 1988. Monoclonal antibodies to sperm surface antigens of the brown alga Fucus serratus exhibit region-, gamete-, species- and genus-preferential binding. Planta 176: 298–306. doi: 10.1007/BF00395410 24220858

[mcaf103-B34] Kloareg B, Quatrano RS. 1988. Structure of the cell walls of marine algae and ecophysiological functions of the matrix polysaccharides. Oceanography and Marine Biology 26: 259–315. doi: 10.1201/9780429050193-2

[mcaf103-B35] Kolb HC, Finn MG, Sharpless KB. 2001. Click chemistry: diverse chemical function from a few good reactions. Angewandte Chemie International Edition 40: 2004–2021. doi: 10.1002/1521-3773(20010601)40:11<2004::AID-ANIE2004>3.0.CO;2-5 11433435

[mcaf103-B36] Lahaye M . 2001. Developments on gelling algal galactans, their structure and physico-chemistry. Journal of Applied Phycology 13: 173–184. doi:10.1023/A:1011142124213

[mcaf103-B37] Lahaye M, Robic A. 2007. Structure and functional properties of ulvan, a polysaccharide from green seaweeds. Biomacromolecules 8: 1765–1774. doi: 10.1021/bm061185q 17458931

[mcaf103-B38] Lahaye M, Rochas C. 1991. Chemical structure and physico-chemical properties of agar. International Workshop on Gelidium 221: 137–148. doi: 10.1007/978-94-011-3610-5_13

[mcaf103-B39] Leandro A, Pacheco D, Cotas J, Marques JC, Pereira L, Gonçalves AMM. 2020. Seaweed’s bioactive candidate compounds to food industry and global food security. Life 10: 8. doi: 10.3390/life10080140 32781632 PMC7459772

[mcaf103-B40] Lim B-L, Ryu I-H. 2009. Purification, structural characterization, and antioxidant activity of antioxidant substance from the red seaweed gloiopeltis tenax. Journal of Medicinal Food 12: 442–451. doi: 10.1089/jmf.2007.0688 19459750

[mcaf103-B41] Lion C, Simon C, Huss B, et al 2017. BLISS: a bioorthogonal dual-labeling strategy to unravel lignification dynamics in plants. Cell Chemical Biology 24: 326–338. doi: 10.1016/j.chembiol.2017.02.009 28262560

[mcaf103-B42] Liu X, Wolfe R, Welch LR, Domozych DS, Popper ZA, Showalter AM. 2016. Bioinformatic identification and analysis of extensins in the plant kingdom. PLoS One 11: e0150177. doi: 10.1371/journal.pone.0150177 26918442 PMC4769139

[mcaf103-B43] Lomartire S, Marques JC, Gonçalves AMM. 2022. An overview of the alternative use of seaweeds to produce safe and sustainable bio-packaging. Applied Sciences 12: 3123. doi: 10.3390/app12063123

[mcaf103-B44] Louis J, Ballu S, Rossi N, et al 2023. Multi-year renewal of green tides: 18 years of algal mat monitoring (2003–2020) on French coastline (Brittany region). Marine Pollution Bulletin 193: 115173. doi: 10.1016/j.marpolbul.2023.115173 37352802

[mcaf103-B45] Makkar HPS, Tran G, Heuzé V, et al 2016. Seaweeds for livestock diets: a review. Animal Feed Science and Technology 212: 1–17. doi: 10.1016/j.anifeedsci.2015.09.018

[mcaf103-B46] Marketsandmarkets . 2023. Algae products market overview 2028. https://www.marketsandmarkets.com/Market-Reports/algae-product-market-250538721.html (11 June 2024, date last accessed).

[mcaf103-B47] Martone PT . 2010. Quantifying growth and calcium carbonate deposition of *Calliarthron cheilosporioides* (Corallinales, Rhodophyta) in the field using a persistent vital stain. Journal of Phycology 46: 13–17. doi: 10.1111/j.1529-8817.2009.00770.x

[mcaf103-B48] Mbua NE, Guo J, Wolfert MA, Steet R, Boons G-J. 2011. Strain-promoted alkyne–azide cycloadditions (SPAAC) reveal new features of glycoconjugate biosynthesis. Chembiochem: a European Journal of Chemical Biology 12: 1912–1921. doi: 10.1002/cbic.201100117 21661087 PMC3151320

[mcaf103-B49] Messyasz B, Czerwik-Marcinkowska J, Massalski A, et al 2013. Morphological and ultrastructural studies on *Ulva flexuosa* subsp. *Pilifera* (chlorophyta) from Poland. Acta Societatis Botanicorum Poloniae 82: 2. doi: 10.5586/asbp.2013.013

[mcaf103-B50] Meyer MT, Whittaker C, Griffiths H. 2017. The algal pyrenoid: key unanswered questions. Journal of Experimental Botany 68: 3739–3749. doi: 10.1093/jxb/erx178 28911054

[mcaf103-B51] Minick M, Fotta K, Khan A. 1991. Polyiodine units in starch–iodine complex: INDO CI study of spectra and comparison with experiments. Biopolymers 31: 57–63. doi: 10.1002/bip.360310106

[mcaf103-B52] Morel O, Lion C, Neutelings G, et al 2022. REPRISAL: mapping lignification dynamics using chemistry, data segmentation, and ratiometric analysis. Plant Physiology 188: 816–830. doi: 10.1093/plphys/kiab490 34687294 PMC8825451

[mcaf103-B53] O’Brien TP, Feder N, McCully ME. 1964. Polychromatic staining of plant cell walls by toluidine blue O. Protoplasma 59: 368–373. doi: 10.1007/BF01248568

[mcaf103-B54] Pattathil S, Avci U, Baldwin D, et al 2010. A comprehensive toolkit of plant cell wall glycan-directed monoclonal antibodies. Plant Physiology 153: 514–525. doi: 10.1104/pp.109.151985 20363856 PMC2879786

[mcaf103-B55] Pereira L . 2018. Seaweeds as source of bioactive substances and skin care therapy—cosmeceuticals, algotheraphy, and thalassotherapy. Cosmetics 5: 68. doi: 10.3390/cosmetics5040068

[mcaf103-B56] Popper ZA . 2024. 13-We All stand together: an exploration of the drivers and constraints that have shaped plant and algal cell wall diversity. In: Geitmann A. ed. Plant cell walls, 1st ed. Boca Raton, FL: CRC Press, 275–297.

[mcaf103-B57] Popper ZA, Michel G, Hervé C, et al 2011. Evolution and diversity of plant cell walls: from algae to flowering plants. Annual Review of Plant Biology 62: 567–590. doi: 10.1146/annurev-arplant-042110-103809 21351878

[mcaf103-B58] Prabhu M, Chemodanov A, Gottlieb R, et al 2019. Starch from the sea: the green macroalga *Ulva ohnoi* as a potential source for sustainable starch production in the marine biorefinery. Algal Research 37: 215–227. doi: 10.1016/j.algal.2018.11.007

[mcaf103-B59] Přerovská T, Henke S, Bleha R, et al 2021. Arabinogalactan-like glycoproteins from *Ulva lactuca* (Chlorophyta) show unique features compared to land plants AGPs. Journal of Phycology 57: 619–635. doi: 10.1111/jpy.13121 33338254

[mcaf103-B60] Pueschel CM, Cole KM. 1982. Rhodophycean pit plugs: an ultrastructural survey with taxonomic implications. American Journal of Botany 5: 703–720. doi: 10.2307/2442960

[mcaf103-B61] Raimundo SC, Avci U, Hopper C, Pattathil S, Hahn MG, Popper ZA. 2016. Immunolocalization of cell wall carbohydrate epitopes in seaweeds: presence of land plant epitopes in Fucus vesiculosus L. (Phaeophyceae). Planta 243: 337–354. doi: 10.1007/s00425-015-2412-3 26411728

[mcaf103-B62] Ropitaux M, Hays Q, Baron A, et al 2022. Dynamic imaging of cell wall polysaccharides by metabolic click-mediated labeling of pectins in living elongating cells. The Plant Journal: For Cell and Molecular Biology 110: 916–924. doi: 10.1111/tpj.15706 35165972

[mcaf103-B63] Ross AG . 1953. Some typical analyses of red seaweeds. Journal of the Science of Food and Agriculture 4: 333–335. doi: 10.1002/jsfa.2740040706

[mcaf103-B64] Rybak AS . 2018. Species of *Ulva* (Ulvophyceae, Chlorophyta) as indicators of salinity. Ecological Indicators 85: 253–261. doi: 10.1016/j.ecolind.2017.10.061

[mcaf103-B65] Rybak AS, Gąbka M. 2018. The influence of abiotic factors on the bloom-forming alga *Ulva flexuosa* (ulvaceae, chlorophyta): possibilities for the control of the green tides in freshwater ecosystems. Journal of Applied Phycology 30: 1405–1416. doi: 10.1007/s10811-017-1301-5 29755209 PMC5928185

[mcaf103-B66] Rydahl MG, Kracun SK, Fangel JU, et al 2017. Development of novel monoclonal antibodies against starch and ulvan—implications for antibody production against polysaccharides with limited immunogenicity. Scientific Reports 7: 9326. doi: 10.1038/s41598-017-04307-2 28839196 PMC5570955

[mcaf103-B67] Schindelin J, Arganda-Carreras I, Frise E, et al 2012. Fiji: an open-source platform for biological-image analysis. Nature Methods 9: 676–682. doi: 10.1038/nmeth.2019 22743772 PMC3855844

[mcaf103-B68] Schwinn MK, Machleidt T, Zimmerman K, et al 2018. CRISPR-mediated tagging of endogenous proteins with a luminescent peptide. ACS Chemical Biology 13: 467–474. doi: 10.1021/acschembio.7b00549 28892606

[mcaf103-B69] Ścieszka S, Klewicka E. 2019. Algae in food: a general review. Critical Reviews in Food Science and Nutrition 58: 3538–3547. doi:10.1080/10408398.2018.1496319 29999416

[mcaf103-B70] Sharmila G, Dinesh Kumar A, Bajhaiya AM, Gugulothu P, Rajesh B. 2021. Biofuel production from macroalgae: present scenario and future scope. Bioengineered 12: 9216–9238. doi: 10.1080/21655979.2021.1996019 34709971 PMC8809944

[mcaf103-B71] Simon C, Lion C, Spriet C, Baldacci-Cresp F, Hawkins S, Biot C. 2018. One, two, three: a bioorthogonal triple labelling strategy for studying the dynamics of plant cell wall formation in vivo. Angewandte Chemie 130: 16907–16913. doi: 10.1002/ange.201808493 30370981

[mcaf103-B72] Stace CA . 1989. Plant taxonomy and biosystematics. Cambridge, UK: Cambridge University Press.

[mcaf103-B73] Stiger-Pouvreau V, Bourgougnon N, Deslandes E. 2016. Chapter 8—carbohydrates from seaweeds. In: Fleurence J, Levine I. eds. Seaweed in health and disease prevention. Boston, MA: Academic Press, 223–274. doi: 10.1016/B978-0-12-802772-1.00008-7

[mcaf103-B74] Subramanian N, Babu Sreemanthula J, Balaji BR, Kanwar J, Biswas J, Krishnakumar S. 2014. A strain-promoted alkyne–azide cycloaddition (SPAAC) reaction of a novel EpCAM aptamer–fluorescent conjugate for imaging of cancer cells. Chemical Communications 50: 11810–11813. doi: 10.1039/C4CC02996H 25005751

[mcaf103-B75] Torode TA, Marcus SE, Jam M, et al 2015. Monoclonal antibodies directed to fucoidan preparations from brown algae. PLoS One 10: e0118366. doi: 10.1371/journal.pone.0118366 25692870 PMC4333822

[mcaf103-B76] Torode TA, Siméon A, Marcus SE, et al 2016. Dynamics of cell wall assembly during early embryogenesis in the brown alga fucus. Journal of Experimental Botany 67: 6089–6100. doi: 10.1093/jxb/erw369 27811078 PMC5100021

[mcaf103-B77] Usov AI . 2011. Polysaccharides of the red algae. Advances in Carbohydrate Chemistry and Biochemistry 65: 115–217. doi: 10.1016/B978-0-12-385520-6.00004-2 21763512

[mcaf103-B78] Vreeland V . 1970. Localization of a cell wall polysaccharide in a brown alga with labeled antibody. Journal of Histochemistry & Cytochemistry 18: 371–373. doi: 10.1177/18.5.371 4912309

[mcaf103-B79] Vreeland V, Laetsch WM. 1989. Identification of associating carbohydrate sequences with labelled oligosaccharides. Planta 177: 423–434. doi: 10.1007/BF00392610 24212484

[mcaf103-B80] Vreeland V, Zablackis E, Laetsch WM. 1992. Monoclonal antibodies as molecular markers for the intracellular and cell wall distribution of carrageenan epitopes in *Kappaphycus* (Rhodophyta) during tissue development. Journal of Phycology 28: 328–342. doi: 10.1111/j.0022-3646.1992.00328.x

[mcaf103-B81] Wahlström N, Nylander F, Malmhäll-Bah E, et al 2020. Composition and structure of cell wall ulvans recovered from *Ulva* spp. along the Swedish west coast. Carbohydrate Polymers 233: 115852. doi: 10.1016/j.carbpol.2020.115852 32059903

[mcaf103-B82] Wichard T, Charrier B, Mineur F, Bothwell JH, Clerck OD, Coates JC. 2015. The green seaweed *Ulva*: a model system to study morphogenesis. Frontiers in Plant Science, 6, 72. 10.3389/fpls.2015.00072 25745427 PMC4333771

[mcaf103-B83] Wu H, Gao G, Zhong Z, Li X, Xu J. 2018. Physiological acclimation of the green tidal alga *Ulva prolifera* to a fast-changing environment. Marine Environmental Research 137: 1–7. doi: 10.1016/j.marenvres.2018.02.018 29478766

[mcaf103-B84] Young CS, Lee C-S, Sylvers LH, Venkatesan AK, Gobler CJ. 2022. The invasive red seaweed, *Dasysiphonia japonica*, forms harmful algal blooms: mortality in early life stage fish and bivalves and identification of putative toxins. Harmful Algae 118: 102294. doi: 10.1016/j.hal.2022.102294 36195420

[mcaf103-B85] Yu Y, Jia X, Wang W, et al 2021. Floridean starch and floridoside metabolic pathways of *Neoporphyra haitanensis* and their regulatory mechanism under continuous darkness. Marine Drugs 19: 664. doi: 10.3390/md19120664 34940663 PMC8703398

[mcaf103-B86] Yuguchi Y, Fujiwara T, Miwa H, Shirakawa M, Yajima H. 2005. Color formation and gelation of xyloglucan upon addition of iodine solutions. Macromolecular Rapid Communications 26: 1315–1319. doi: 10.1002/marc.200500283

[mcaf103-B87] Zachleder V, Brányiková I. 2014. Starch overproduction by means of algae. In: Bajpai R, Prokop A, Zappi M. eds. Algal biorefineries. Dordrecht, Netherlands: Springer Netherlands, 217–240. doi: 10.1007/978-94-007-7494-0_9

[mcaf103-B88] Zhang Y, He P, Li H, et al 2019. *Ulva prolifera* green-tide outbreaks and their environmental impact in the Yellow Sea, China. National Science Review 6: 825–838. doi: 10.1093/nsr/nwz026 34691936 PMC8291432

[mcaf103-B89] Zhang F, Li Z, Yin L, et al 2021. ACE2 receptor-modified algae-based microrobot for removal of SARS-CoV-2 in wastewater. Journal of the American Chemical Society 143: 12194–12201. doi: 10.1021/jacs.1c04933 34291944

[mcaf103-B90] Zhang LX, Zhang N, Li J, Wang Z. 2013. New α-glucosidase inhibitory polysaccharides isolated from marine green algae *Enteromorpha linza*. Advanced Materials Research 634–638: 1010–1015. doi: 10.4028/www.scientific.net/AMR.634-638.1010

[mcaf103-B91] Zhao Q, Nakashima J, Chen F, et al 2013. *LACCASE* is necessary and nonredundant with *PEROXIDASE* for lignin polymerization during vascular development in *Arabidopsis*. The Plant Cell 25: 3976–3987. doi: 10.1105/tpc.113.117770 24143805 PMC3877815

