# Supplementary figures and images for: Click & sea: using bioorthogonal click chemistry to visualize seaweed cell walls
Source: Ann Bot. 2025 Jun 10;136(2):437–50. doi: 10.1093/aob/mcaf103 (PMC12445872; doi:10.1093/aob/mcaf103)

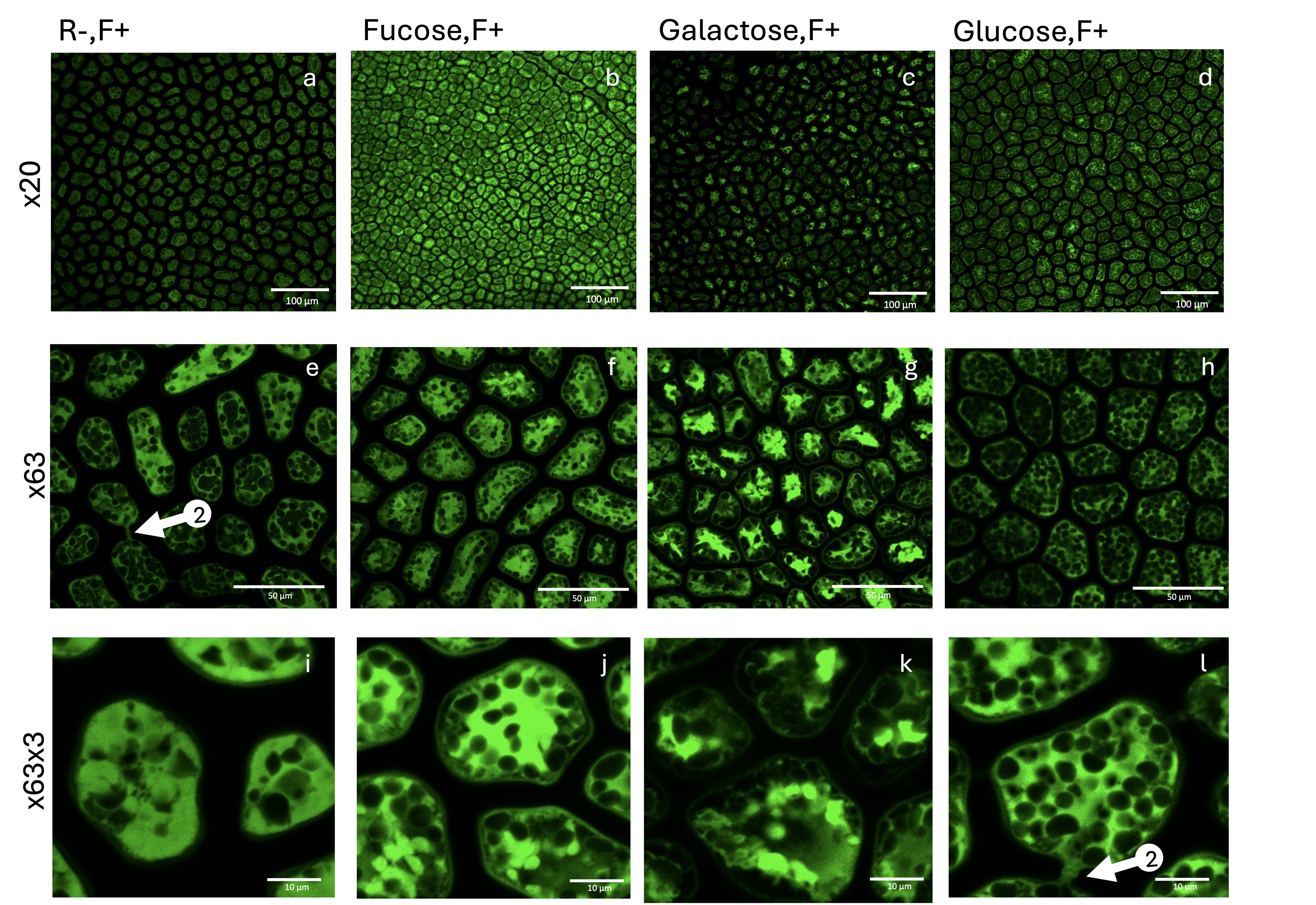

Supplement: mcaf103_Supplementary_Data [file mcaf103_supplementary_data.zip › aob-25009-s02.jpg]

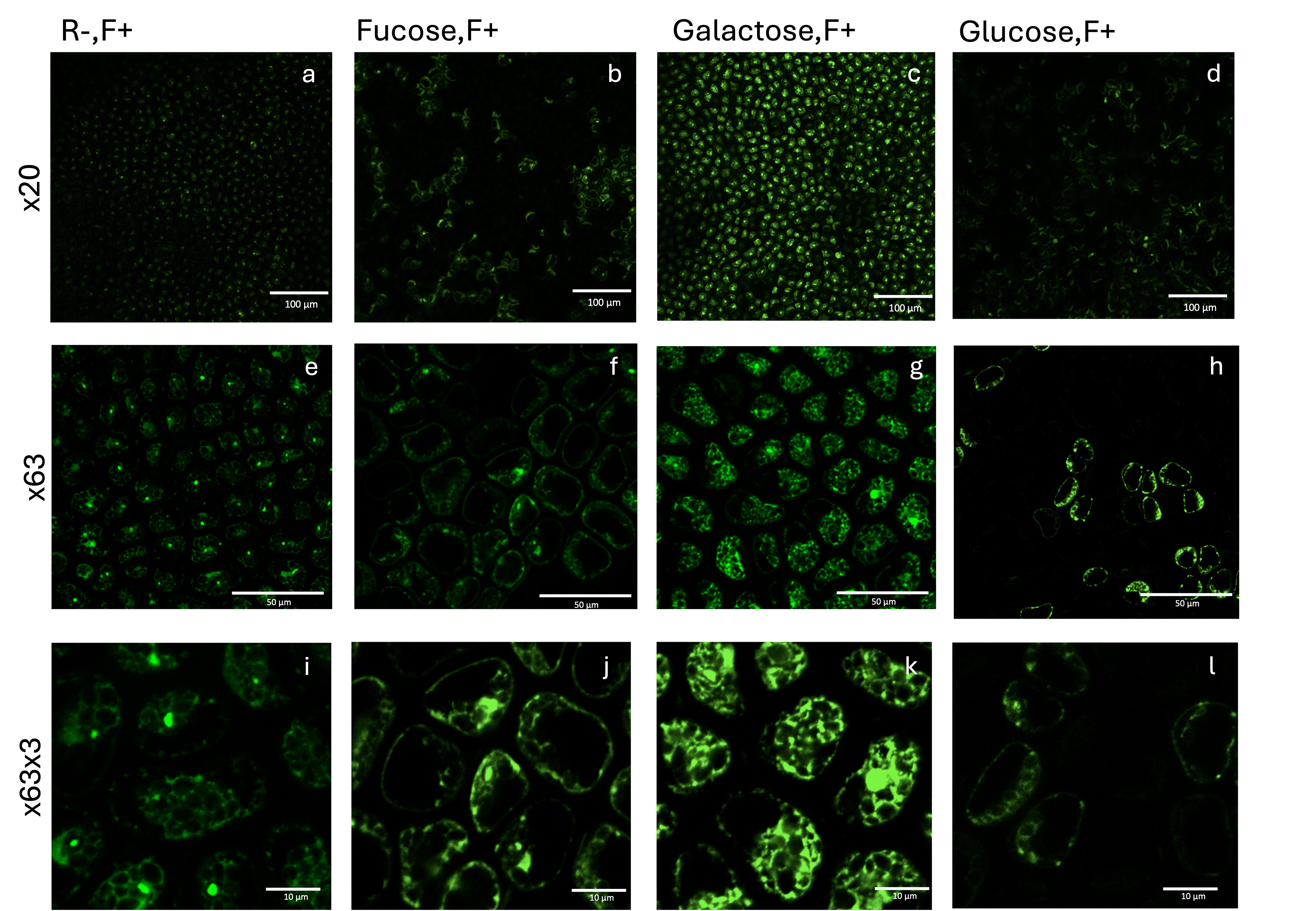

Supplement: mcaf103_Supplementary_Data [file mcaf103_supplementary_data.zip › aob-25009-s03.jpg]
