# Supplementary material for: Click & sea: using bioorthogonal click chemistry to visualize seaweed cell walls
Source: Ann Bot. 2025 Jun 10;136(2):437–50. doi: 10.1093/aob/mcaf103 (PMC12445872; doi:10.1093/aob/mcaf103)
Supplement: mcaf103_Supplementary_Data [file mcaf103_supplementary_data.zip › aob-25009-s01.docx]

Botany and Plant Science,

and Ryan Institute for Environment, Marine and Energy Research,

School of Natural Science,

College of Science and Engineering,

University of Galway,

Galway, Ireland, H91 TK33

[wendy.delpont31@gmail.com](mailto:wendy.delpont31@gmail.com)

+33 637243141

May 6^th^, 2025

Dear Professor Rowan Sage,

On behalf of myself, Wendy F. Delpont, and my co-authors, Godfrey Neutlings and Zoë A. Popper, I would like to resubmit our manuscript, titled “Click and Sea: using bioorthogonal click chemistry to visualise seaweed cell walls” as a technical article for publication in Annals of Botany. The previous manuscript submission code was 25009, and the handling editor was Gitte Petersen.

We would like to thank the handling editor and reviewers for their time, critique and comments. We have taken these into careful consideration in revising our manuscript. We have addressed the comments as detailed in our rebuttal below in red and revised our manuscript accordingly using track changes in the revised uploaded manuscript.

Your sincerely,

Wendy F. DELPONT

**Referee #1's Review for 25009:**

Comments for the Author (Required): The manuscript entitled, "Click & Sea: using bioorthogonal click chemistry to visualise seaweed cell walls" by Delpont, Neutelings and Popper, describes the methods used for applying click chemistry technology for analyses of the cell walls of one red and one brown alga. This technique uses chemically-modified monosaccharides (i.e., chemically activated sugars, glucose, galactose and fucose) and fluorescent probes to image cell wall polysaccharides/glycoproteins and potentially, their biosynthetic pathways. This technique has been used in studies of plant cell walls and in this project, is applied to seaweeds for the first time.

1) The paper is well-written. While somewhat long, it provides necessary detail and sufficient background for the reader to understand this newer technology. The photographic images, graphs and diagram are well-composed and provide adequate data for subsequent interpretation of the applicability and future of click chemistry in seaweed cell wall studies. It is also nice that the authors provided good descriptions of the cell walls of seaweeds from different taxonomic groups. This will assist readers not familiar with algal extracellular coverings.

2) The authors make a good case for why this technology is needed as well as its potential benefits when compared to previous immunocytochemical and biochemical technologies. The scope of this paper will be of significant interest to cell wall biologists, phycologists and those in applied industries using seaweeds today and in the future.

3) There are a couple of minor points that need to be addressed:

1) what happens if one does not use 70% methanol for washing? I assume that there is residual fluorescence from the fluorophore that's not previously removed. But, doesn't this step the eliminate subsequent in vivo studies?;

Details added. The need to use methanol (Simon et al., 2018; doi:10.3791/56947) or ethanol (Pandey 2016; doi:10.3389/fpls.2016.01309) to remove fluorophores has been shown previously in terrestrial plants and is indeed crucial for reducing background on large sized samples. This treatment does prevent further studies under in vivo conditions, but these are not necessary in our case.

2) While there are few antibodies raised against seaweed wall polymers, why not use one or two for comparison with results obtained by click chemistry?;

Details added (page 7, line 22), antibodies are no longer available commercially. Also, even if the comment is relevant, it should be remembered that the click approach enables us to estimate the potential of the cell walls to incorporate reporters corresponding to the targeted monomers, whereas antibodies will detect polymers that have already been synthesized. Each tool is therefore able to answer different biological questions.

3) Click chemistry has been advertised quite a bit and has been demonstrated as a novel and potentially important technique for plant cell wall studies. Yet, somehow its use has been notably limited. Why is this? Would it be possible to add a statement or two commenting on this technology. Is it too difficult a technique? too expensive or difficult to prepare the reagents? How does this seaweed study then aid researchers in considering the use of this technology?

Details added (page 24, line 4: Click chemistry is a technique with numerous advantages: it is relatively simple and inexpensive to perform. However, its interpretation is more complex than that of monoclonal antibodies, as the activated sugar can be integrated into various components of the seaweed. While monoclonal antibodies recognize specific epitopes in polymers, their use is more limited. One advantage of click chemistry over antibody staining is its application in cases where polymers are not well-defined (Popper, 2024).

This method opens many avenues of research, including determining changes in seaweed cell walls related to the life cycle and various stresses (grazing, pathogen infection, etc.) that could impact seaweed ecology and diversity, as well as the quality of commercially grown seaweed and the products derived from them).

Also the limit to the use of click chemistry is certainly the availability of the reporters. In this work, tag-bearing monosaccharides were obtained from commercial sources, but in other cases, as with monolignols, they need to be synthesized. So, for the study of algal walls, it's relatively easy to use this approach, since they contain polymers of glucose, galactose, fucose or even mannose all of which are commercially available in conjugated forms.

We've added a sentence to page 8 line 21-25 of the introduction to argue these points: The use of click chemistry approaches is generally straightforward from a technical point of view, with the only possible difficulties being the mobility of reporters across cell interfaces and verification of their compatibility with cellular metabolism. The real challenge, however, remains their synthesis when they are not commercially available, as is the case for lignin precursors (Morel et al., 2022).

4) In overview, this is a very nice paper that will significantly add to plant/algal cell studies.

Thank you

**Referee #2's Review for 25009:**

Comments for the Author (Required): This MS demonstrates the feasibility of using bioorthogonal click chemistry to image cell wall components in seaweeds. This is technically skilled work that has significant potential. However, the analysis of images could be more precise and the biological novelty of the findings could be stressed more strongly. For example, the authors have 24h and 48h uptake data for their click chemistry: can their results be used to test the hypothesis that different reporters are incorporated into the cell wall at different rates?

The 24H and 48H time-points were chosen to ensure optimal incorporation of the various reporters. If we wanted to quantify the incorporation kinetics of each reporter in detail, we would have to consider independent labelling of each of them individually within the same sample. This is what we have down in the context of lignification, by using a triple-click approach. However, it will be necessary to synthesize reporters bearing 2 additional tags (alkyne and methylcyclopropene groups) as only the versions with azide functions are commercially available.

Alternatively, could the imaging data be more rigorously analysed to test the hypothesis that localisation patterns are different between reporters (at the moment, the localisation analysis is subjective)?

Same answer as above, yes absolutely only if 3 different tags are combined to 3 different reporters. We have previously shown that it was possible to detect with a confocal Airyscan microscope, differential fluorescence associated with monolignol surrogates in cell wall substructures, such as pits or in wall layers that are otherwise not distinguishable by classical LSCM (Simon et al., 2023; https://doi.org/10.1021/cbmi.3c00052)

- Abstract

This an accurate summary of the main text.

Thank you

- Introduction

The introduction is generally good, although there are a couple of sentences that are ambiguous p.4 l.10 makes it sound as though Sargassum can be 70m long, rather than seaweeds?

Rephrase (page 4, line 9: Some seaweeds can be very small, couple of cm, while can be up to 70 meters long.)

The segue from structural polymers to storage polymers is abrupt.

Rephrase (page 5, line 4: Carbohydrates are stored as starch in green seaweed, like in land plants. This starch is formed of two polymers, amylose and amylopectin, composed of D-anhydroglucopyranose (Prabhu et al., 2019).

The paragraph starting p. 6, l. 6 may make more sense towards the start of the introduction).

We believe it is better when the Introduction goes from the seaweed in general to the cell walls, this is because we went to give a general introduction for a broad audience of the test subject i.e. seaweed that is novel for click chemistry. Click chemistry has been used to explore plant cell wall so it is then a comparator in this case.

There are some typos (p.4, l.23: 'glucoronate';

Corrected

p.7 l.8-10 grammar needs attention).

Rephrased

Page 7 line 4-10: A few monoclonal antibodies that were developed against land plant cell wall components can recognise features present in brown (Raimundo et al., 2016) and green seaweeds (Estevez et al. 2009; Fernández et al. 2010). However, the development of highly specific monoclonal antibodies that label structures present in seaweed cell wall polysaccharides, giving insight into their location and metabolism, has been very limited. This is especially the case in comparison to the large array of different monoclonal antibodies available against plant cell wall components (Duffieux et al., 2020; Pattathil et al., 2010).

- Methods

Again, some typos (p.10, l.4-5: no 'e' at the end of '-phyta'; p.13, l14: correct 'Phycrodrys').

Corrected

Quantification in Table 2 and Figure 6: how exactly was this done? Many of the pixels in Fig 5 seem to be saturated? (See also comment on legend to Fig 6, below)

Table 2 shows the main trends (not quantified) that can be observed overall in the images. The quantification (figures 5 & 6) was performed by measuring the signal intensities in FIJI. It was first assessed by determining a mean background threshold on R-F+ samples. The determined signal background was then subtracted from signal values measured along algal cell walls that are clearly detached from the intracellular fluorescence.

It is important to point out that the settings made during image capture on the confocal microscope (all identical for the same series to be compared) ensure that no pixel is saturated. This is important for subsequent quantification.

- Results: These are novel and technically demanding. The patterns of staining are also interesting. However, their description and interpretation would benefit from more clarity, as below:

For the description of results, the text and figures could work better together (eg. p.14, l. 11: perhaps add that this is in Fig 5 as well?

We have now included references to figure 5 and 7 in the text. This figures collate labelling images with different activated sugars residues for the different seaweeds.

p.16, l.1-3: the description here doesn't match Fig 6? For example, median intracellular Fuc is higher than median intracellular Glc, Fuc is higher than Gal in cell walls, Fuc is higher intracellularly than in cell wall?

The comments in this paragraph concerns only Phycodrys. As shown in Figure 6, in this species, glucose incorporation is higher in both compartments and the signal corresponding to galactose is higher in the cell wall compared to fucose but the opposite is shown in the intracellular compartment.

p.21, l.9-12: Arrow 3 in Fig 9e is identified as being a pyrenoid, but in the Figure legend to 9e, it's identified as the nucleus. Which is it?

Corrected

p.22, l.11: Is Fig 7l the fluorochrome control, rather than 7i?)

Corrected

- There are also some questions around the interpretation of results:

p.16, l.20: Approximately three brown-staining spots per cell? How was this calculated? Fig 9e makes it look like there are several times that number?

We count 14 cells and did an average, result average was 3,38

p.19, l.24 (and Fig 8e): What is the evidence for this structure being the pyrenoid, rather than the nucleus?

Changed in the figure legend and text page 21 line 8.

p.20, l.4: The Iodine staining seems to agree better with Supp Fig 1l?

Supp Fig 1l added in the text page 20, line 1

p.20, l.5-6 states that the glucose reporter is likely to bind starch and, naively, one might expect the Glc reporter to become incorporated into starch granules. However, that doesn't seem to be the case in Figs 5 and 7 - why not?

Sentence added page 20, lines 2-4: However, it can be observed that glucose labelling in starch grains is not as bright as expected. This could be due to a stressful environment for the cells, affecting starch production (Zachleder & Brányiková, 2014).

Page 20, lines 9-11: In Ulva, punctate fluorescence in the cell walls may be due to localized differences within the cell wall, resulting in the observed fluorescence pattern.

For Ulva, there seems to be punctuate fluorescence in the cell walls ('bright spots') that aren't present in Phycodrys. Are these artefacts?

The punctuate fluorescence observed for *Ulva* cell walls are likely to be derived from localised deposition of cel wall polymers; either of those containing the fluorescent monomer or polymers that block for the fluorescence.

There is evidence that seaweed cell wall polysaccharides can be localised e.g. monoclonal antibodies CCRC_M26 labelled Fucus vesiculosus sperm inner walls and egg mesochite (Raimundo et al 2016 https://doi.org/10.1007/s00425-015-2412-3). Punctate labelling of cell wall components has been observed e.g. for pectin and other components in plant pollen tubes (Moussu et al 2023, DOI: 10.1126/science.adi4720).

For Phycodrys (and for some Ulva samples), it is hard to see any difference in localisation patterns between reporters, although there are some possible trends (e.g. the R-F+ seem to have less wall staining in the 63x images, there seems to be some pit plug staining [Fig 7i, m] in some cases [although in the R-F+ as well, Supp Fig 2, e],

Sentence added

Page 21, line 11-15: Pit plugs are also visible using fucose and galactose reporters in click chemistry Fig. 7i, m, Supplementary Fig 2e (arrow 2).

Based on a comparison of Supp Fig 2, j & l with Fig 7m & o, the label seems to move from cytosolic after 24h to internal compartments and cell wall by 48h, etc etc.)

The 24H and 48H time-points were chosen to ensure optimal incorporation of the various reporters. If we wanted to quantify the incorporation kinetics of each reporter in detail, we would have to consider independent labelling of each of them individually within the same sample. This is what we have down in the context of lignification, by using a triple-click approach. However, it will be necessary to synthesize reporters bearing 2 additional tags (alkyne and methylcyclopropene groups) as only the versions with azide functions are commercially available.

In land plants transit time from Golgi to cell wall for polysaccharides is approx. 20 minutes (Fry, 2004 https://doi.org/10.1111/j.1469-8137.2004.00980.x; Northcote and Pickett-Heaps, 1966 https://doi.org/10.1042/bj0980159). Therefore, it is likely to get an accurate idea of metabolism and incorporation into the various cellular compartment, more and earlier time points would have needed to be investigated. These two time points, 24- and 48hours, were used as preliminary test of incorporation only.

Have any of these observations (or others) been tested as hypotheses? Fiji has a lot of functionality around image pattern analysis - particle analysis, threshholding etc - was any of that used?

No we have not tested the hypothesis of label movement within the framework of this project.

Finally, there are some typos (e.g. p.21, l.4: is unfinished; p.20, l.15: pit plugs probably are analogous to plasmodesmata because they do the same thing, but they're not thought to have descended from the same ancestral structure, so aren't thought to be homologous)

Sentence rephrase

Page 21, line 13-15: Pit plugs facilitate cell-cell communication but are not considered homologous to plasmodesmata, which are found in land plants (Pueschel & Cole, 1982). However, despite their different origins, they perform similar roles.

- Tables and Figures

Table 1

Check spelling of carrageenan.

Corrected

Table 2

Why not include R-F-?

The possible autofluorescence (only present in *Phycodrys*) is already included in the R-F+ signal in addition to the low aspecific background signal due to the fluorescent probe.

Figs 5, 7-9

The labels on the scale bars are very small. Also, do the 20x images provide any information that isn't in the 62x images?

Scale bars changed

Fig 6: How many data points make up each distribution in these box-and-whisker plots and where exactly do the data points come from? Does each data point represent a single cell in the same thallus? If so, how were cells chosen? Also if so, would multiple cells from a single thallus be considered biological replicates?

As mentioned above (methods, Quantification in Table 2 and Figure 6), for the cell walls, each data point comes from a measure performed on a portion clearly detached from the intracellular fluorescence. If the signal in the cell wall and the intracellular compartment where to close, the portion of the cell wall were not considered. The box-and-whisker plots represent at least 11 measurements. Considering individual cells as biological replicates does not seem relevant from our point of view.

- Legends

p.34, l.6: insert 'azido' into the fucose reporter (also p.35, l.7)

Corrected

p.35, l.10: Should be Fig 3?

Corrected

p.35, l.16: correct 'biorthogonal'

Corrected

p.35, l.21 (and passim): I'm guessing the x3 in x63x3 isn't true magnification, but digital zoom? Perhaps call it x63 (expanded), vel sim.

Detail added

Page 35, lines 21-22, page 36, lines 12-13: (x63 expanded three time)

Figs 5, 7: Specify what the arrows indicate (cell wall?).

Details added:

Page 36, line 1-2: The arrows indicate the cell walls, line 16: The arrows 1 indicate the cell walls, arrows 2 shows the pit-plugs.

Fig. 6; What units are used for fluorescence (AU?)? Exactly what fluorescence is being measured: total in the cell? Average in the cell? Average in labelled areas?

In FIJI, fluorescence was given in arbitrary units (AU). It measures average gray value (the colour on the images are false colours) within the selection. This is the sum of the gray values of all the pixels in the selection divided by the number of pixels.

We added the label AU in the y-axis in Figure 6.
